# Evaluating the adoption of handsearching, citation chasing, and screening tools in education research: a survey study

**DOI:** 10.3389/frma.2024.1430355

**Published:** 2024-11-27

**Authors:** Qiyang Zhang, Marta Pellegrini, Francesco Marsili, Amanda Neitzel

**Affiliations:** ^1^Johns Hopkins University, Baltimore, MD, United States; ^2^Department of Pedagogy, Psychology, Philosophy, University of Cagliari, Cagliari, Italy; ^3^Department of Philosophy, Social and Human Sciences and Education, University of Perugia, Perugia, Umbria, Italy; ^4^School of Education, Johns Hopkins University, Baltimore, MD, United States

**Keywords:** systematic reviews, meta-analysis, survey, screening tools, citation searching tools, handsearching tools, education

## Abstract

**Introduction:**

The rapid development of software tools to assist systematic reviewers has led to varying degrees of adoption and selection among researchers. However, the actual usage patterns of these tools, their preferred features, and the criteria for selecting the most suitable tools remain unclear.

**Methods:**

To understand these aspects, we collected 175 responses from researchers across different continents.

**Results:**

In terms of handsearching, despite new tools developed, our findings reveal that manual handsearching remains prevalent among more than half of the participants. Databases are the most popular tools for citation searching, followed by citation management tools and spreadsheets. This reliance on citation management tools and spreadsheets is concerning as they are not specifically designed for systematic reviews. The primary factors influencing tool selection are the research environment and ease of use. Barriers stopping researchers from adopting alternative tools include limited awareness, challenges in learning new tools, and the financial costs associated with acquiring licenses. Moreover, researchers located in Europe show greater familiarity with a wider range of tools compared to their North American counterparts.

**Discussion:**

This preregistered study contributes valuable insights into the tool usage patterns of education researchers, emphasizing the importance of promoting awareness and facilitating the broader adoption of existing tools.

*If a workman wishes to do a good job, he must first sharpen his tools [*工欲善其事, 必先利其器*]—Confucius*

## 1 Introduction

Systematic reviewers using tools is similar to craftsman honing tools for a meticulous task. Over the past two decades, numerous software tools have emerged to streamline the searching and screening stages of reviews, replacing manual processes with semi-automated ones. While use of these tools is growing among educational researchers, their reach is far from universal (Zhang and Neitzel, 2024). Given how these tools can streamline research synthesis, there is a lack of understanding regarding their preferred tools and the factors influencing these choices and preferences. What do researchers think of the existing tools? Which tools are they using or planning to adopt in the future? Which tools do they prefer? Does this pattern differ by continents or demographic characteristics? What are barriers preventing them from selecting alternative tools? This study aims to explore the current usage of searching and screening tools in educational systematic reviews by collecting and reporting survey results.

### 1.1 Why is tool usage important in systematic reviews?

Systematic reviews are vital research products that synthesize primary study findings to provide evidence for research, policy, and practice. They help identify research gaps and support evidence-based decision-making (Cooper et al., 2019; Slavin, 2008). The quality of systematic reviews is crucial. Guidelines like PRISMA outline requirements for transparent^1^ and reproducible reporting (Alexander, 2020; Page et al., 2021; Pigott and Polanin, 2020). The quality of evidence (e.g., rigor and recentness) directly influences its impact on policy (Davies, 2000; Ming and Goldenberg, 2021). However, these quality factors of systematic reviews are often contingent upon the methods employed, including in the domains of handsearching, citation chasing, and reference screening processes. In a time when trust diminishes with the aging of evidence, it is crucial to conduct reviews swiftly, yet thoroughly and transparently. It is hard to convince policymakers to trust and ground their decisions on systematic reviews based on articles retrieved 5 or 10 years ago. This becomes especially pertinent when considering the laborious nature of information retrieval, the ever-expanding body of research work, and the reliance on up-to-date evidence. For instance, on average, a systematic review project requires five researchers and 67.3 weeks to complete (Borah et al., 2017). With help from automation tools, the process of completing a moderate-sized review can be shortened from 1.5 years to just 2 weeks (Clark et al., 2020). To influence policy and practice effectively, high-quality systematic reviews must be conducted with both reliability and speed.

Reliability and speed are essential, but equally critical is the assurance that no vital information is overlooked. The proverb “garbage in, garbage out” highlights the importance of the informal retrieval tools used in the systematic review process. Searching the literature and screening studies are two key stages to build a strong foundation for a quality systematic review. A comprehensive literature search includes more than a search of academic databases. It also includes handsearching and citation chasing, which are further explained below. Low-quality handsearching and citation chasing processes may lead to missing relevant studies, while low-quality study screening and selection processes may lead to human errors and a lengthy process (Haddaway et al., 2022; Zhang and Neitzel, 2024). Technological tools specifically developed for these stages may support the reproducibility of the review and the reliability of its results. In meta-analyses, while statistical analyses receive due attention, the tools instrumental in the review process remain in the shadows. There is a dearth of research focusing on how to conduct systematic reviews in practice and what tools to use in data collection and extraction. This is concerning because when reviewers employ inefficient methods in the data collection phase, they could potentially compromise the rigor of the entire systematic review. For example, a typical search string may return thousands of results. Using a tool to support screening will result in a transparent and replicable way to screen those results. The alternative may include individuals “eyeballing” results within the search results, recording those that appear relevant, with no history of decisions and reasons recorded. Another instance is when researchers used to conduct handsearching by physically flipping through countless volumes of journals and books to scan tables of contents to identify relevant literature in libraries. While the more modern version includes reading electronic tables of contents, this practice still results in a lack of transparency and documentation of how studies were determined to be included or not. These practices are time-consuming, prone to oversight, and highly inefficient.

In today's digital age, where typewriters have evolved into collaborative online platforms with grammar and spelling checking, the methodologies in systematic reviews demand a similar upgrade. Understanding the tools used and their impact is not just an academic exercise; it directly influences the credibility and relevance of systematic reviews in providing evidence for policymaking. Many tools have been recently developed; however, little is known about the actual usage of these tools among reviewers, about which characteristics are preferred, and how to choose the most appropriate tool. Furthermore, the global landscape of tool production introduces an additional layer of complexity. Tools are predominantly produced in certain continents, such as North America and Europe, prompting questions about potential regional variations in usage patterns and their implications for the quality of systematic reviews. To broaden access and enhance global collaboration, it is crucial to investigate and understand these usage patterns across continents.

### 1.2 Insights on tool usage from previous studies

Previous studies have compared the usability and features of screening tools in non-educational fields (Harrison et al., 2020; Van der Mierden et al., 2019). Van der Mierden et al. (2019) analyzed the features of commonly used tools in biomedical research and found that DistillerSR, EPPI-Reviewer, Covidence, and SWIFT Active Screener possess all the necessary features for conducting the screening phase. They rated Rayyan as the top-performing free software, while tools not specifically designed for systematic reviews (e.g., Microsoft Word and EndNote) received lower scores. Harrison et al. (2020) conducted a similar analysis of user survey in healthcare and recommendied Covidence and Rayyan as suitable tools for systematic reviews.

In the field of education, Zhang and Neitzel (2024) conducted one of the pioneering studies addressing this issue by examining explicit mentions of screening tool usage and investigating their features. They discovered that only 4.19% of the studies published in *Review of Educational Research* reported using a screening tool. The lack of reporting on software tools for the screening process suggests that authors either did not use any software or used a tool but deemed it irrelevant to report. Given the lack of transparency in social sciences, such as psychology (Polanin et al., 2020), the limited transparency in reporting tool usage in published articles is not surprising. However, this lack of reporting does make it challenging to assess overall tool usage patterns through secondary data analysis.

Therefore, our motivation in this paper was to collect primary data by surveying educational systematic reviewers worldwide at different stages of their academic careers to better understand their use of these tools beyond what is reported in published studies. This approach allowed us to gain insights into tool usage in educational systematic reviews, promote knowledge about these tools, and encourage researchers to try new tools.

Our research was motivated by two primary factors. Firstly, we observed a lack of tool reporting in published education review articles, which poses challenges for replication (Zhang and Neitzel, 2024). We aimed to investigate whether this low reporting rate is due to a low adoption of tools or simply a habit of not reporting tool usage. Secondly, we recognized that using tools can significantly change the human resources required for the searching and screening stages. This survey aimed to encourage researchers to adopt tools and emphasize the importance of reporting tool usage to enhance the transparency of future systematic reviews.

## 2 Handsearching and citation chasing tools

In addition to database searching, handsearching and citation chasing are two supplementary techniques that help to fetch missing but relevant records in a systematic review (Cooper et al., 2018). Handsearching consists of two steps: (1) identifying a list of the most relevant and high-impact journals and conferences, and (2) browsing through the tables of contents of each journal issue and conference program. Citation chasing, also referred to as snowball searching, can be distinguished in two directions: backward citation chasing and forward citation chasing. Backward citation chasing involves retrieving and assessing the relevance of the records listed in the bibliography of one or more identified articles. Forward citation chasing involves locating records that have cited a specific article or a set of articles.

Traditionally, these supplementary searching techniques are conducted by reviewers who manually search journal websites, online conference programs, reference lists of relevant studies as well as using bibliographic data platforms (e.g., Web of Science, Google Scholar). These traditional ways to conduct handsearching and citation chasing often result in an inefficient and costly procedure for two reasons. First, they are time-consuming because researchers have to export citations individually rather than downloading records in bulk. Second, they only support one direction of citation searching (Pallath and Zhang, 2023).

In the past 5 years or so, several software tools, such as *SpiderCite* (Institute for Evidence-Based Healthcare, 2021), *CoCites* (Janssens and Gwinn, 2015), and *Citation Chaser* (Haddaway et al., 2022), were developed specifically for citation chasing. However, *CoCites* draws data from the United States National Institutes of Health, which limits the content to public health related literature. *Citation Chaser*, the most advanced citation chasing tool, is a free-to-use, open-source tool for conducting rapid backward and forward citation chasing with an accompanying Shiny app. In terms of handsearching, the first tool was developed in 2022. The tool, called Paperfetcher (Pallath and Zhang, 2023), automates both handsearching and citation chasing. These tools can streamline handsearching and citation chasing efforts to produce a comprehensive search with an efficient use of resources.

## 3 Screening tools

After a comprehensive search of the literature, the next stages of title and abstract screening and full-text review are essential to ensure the inclusion of relevant and eligible studies. Selection process can be a particularly laborious and demanding step (Lefebvre et al., 2022). Depending on the review objectives and fields, the number of records to be screened can range considerably from hundreds to thousands. Moreover, the time needed to screen a title and abstract can vary significantly from a few seconds to 1 min based on the way information is presented and the experience of the reviewers (Wang et al., 2020). Guidance papers suggest that each abstract should be independently screened by at least two reviewers, which means that more time is needed to complete this task with best practices (Polanin et al., 2019).

Before specialized screening tools were released, the only option for screening was through spreadsheets or citation management software (e.g., Zotero). However, both tools have evident limits: spreadsheets are not designed for literature review and create various challenges in blind reviewing, managing screening progress, and resolving conflicts, and tracking search details; citation management software provide a more convenient platform for storing and retrieving literature, but still lack essential features for systematic reviews such as tracking history, plotting PRISMA flow chart, and calculating inter-rater reliability.

Recently, the stage of title and abstract screening has also been impacted by the rapid expansion of Artificial Intelligence (AI), which has rapidly gained prominence across the globe. The growth in AI and machine learning applications inspired the development of software tools specifically designed to expedite the screening process. These tools present studies with the highest probability to be included at the beginning of the list based on the records already screened by the team. This feature saves a tremendous amount of resources, which subsequently increases efficiency and reviewers' motivation (Blaizot et al., 2022). The use of these tools is also suggested as a best practice by several guidance papers (Pigott and Polanin, 2020; Polanin et al., 2019). Table 1 shows the main features of existing handsearching, citation searching, and screening tools collected by authors. In this research, we will discuss Citationchaser (Haddaway et al., 2022), CoCites (Janssens and Gwinn, 2015), Paperfetcher (Pallath and Zhang, 2023), SpiderCite (Institute for Evidence-Based Healthcare, 2021), Abstrackr, ASReview, Covidence, EPPI-Reviewer, DistillerSR, Rayyan, and RevMan. These tools were used to design the survey. A narrative description of these tools included in our survey is provided in the Supplementary material.

**Table 1:** Basic information on the available handsearching, citation chasing, and screening tools.

**Name**	**Info**	**Year of launch**	**Developer publication**	**Developer**	**Country**	**Price**
**Handsearching and citation chasing tools**						
Citationchaser	Open R package and web-based Shinny app for citation chasing	2022	Haddaway et al.	-	Germany	Free
CoCites	Online citation-based search tool	2015	Janssens and Gwinn	-	USA	Free
Paperfetcher	Open source Python package and web-app for handsearching in journals and citation chasing	2022	Pallath and Zhang	-	USA	Free
SpiderCite	Open tool included in the Systematic Review Accelerator suite for citation chasing	2022	Institute for Evidence-Based Healthcare	Institute for Evidence-Based Healthcare	Australia	Free
**Screening tools**						
Abstrackr	Semi-automated tool for teamwork in meta-analysis	2012	Wallace et al.	Tufts Evidence-based Practice Center, maintained by Brown Center for Evidence Synthesis in Health	USA	Free
ASReveiw	Uses the latest machine learning algorithms to minimize errors and maximize accuracy	2021	van de Schoot et al.	Utrecht University	Netherlands	Free
Covidence	Web-based systematic review tool for screening, data extraction, and analysis	2013	-	Australian non-profit company Veritas Health Innovation	Australia	Not free $240–$635
EPPI-Reviewer	Supports study screening through data collection, analysis and synthesis Includes features such as text mining, data clustering, classification, term extraction, and machine learning	2010	Thomas et al.	EPPI-Centre at the Social Science Research Unit at the Institute of Education, University College London, and University of London	UK	Not free $144.54–$505.89 and more
DistillerSR	Automates literature collection, triage, and assessment using AI and intelligent workflows	2018	-	Evidence Partners Inc. Ottawa	Canada	Not free $239.4–$3,636
Rayyan	Accelerates abstract/title screening with semi-automation	2014	Ouzzani et al.	Qatar Computing Research Institute: Rayyan Systems Inc.	Qatar	Free with limited functions, $48–$99 and more
RevMan Web/RevMan 5	Facilitates protocol development, screening, and full-text reviews Present the results graphically	2008	The Cochrane Collaboration	The Cochrane Collaboration	UK	$72.87–$120.24 and more

These data were retrieved in 2023 and the prices might shift over years due to adjustment or inflation (Carey et al., 2022; Ouzzani et al., 2016; Review Manager, 2014; Thomas et al., 2020; van de Schoot et al., 2021; Wallace et al., 2012).

## 4 Current study

This study aims to understand the usage patterns of searching and screening tools among systematic reviewers in education as well as the reasons for their preferences. To the best of our knowledge, this is the first survey study on tool usage status in education systematic reviews. In this preregistered^2^ study, we seek to answer the following research questions with the related hypotheses.

Q1: Which software tools are most frequently used to conduct handsearching, citation chasing, and screening in educational systematic reviews?

H1: According to Zhang and Neitzel (2024), among published review papers in education, there is an extremely low rate of reporting tool usage. We hypothesize that systematic reviewers in education use DistillerAI, EPPI-Reviewer, Covidence, and Abstrackr (note that Zotero and spreadsheet do not count as screening tools). In terms of handsearching tools, since these tools were developed only in very recent years, we hypothesize that researchers either skip this search step or manually conduct it. As for citation searching tools, we hypothesize that researchers have not heard about most of the new tools since they were developed recently.

Q2: What are the reasons behind systematic reviewers' choices in software? What are the barriers preventing them from choosing other tools?

H2: Since training teams to adapt to a new software takes time and efforts, we hypothesize that the top two reasons for researchers to not choose certain tools are (1) researchers find it hard and time-consuming to change pre-existing practices, such as screening in spreadsheets, and (2) the high cost of the software license since cost acts as the barrier for academic researchers to access tools. The overall preferred tool must be easy to use and overall least-selected tools must be difficult to use, such as tools that require Python knowledge.

Q3: Do researchers' attitude toward AI-assisted screening tools differ by positions (faculties or doctoral students) and continents?

H3: PhD students' attitude toward AI-assisted software is more accepting compared to faculties. Since faculties are, on average, older than students, there may exist a gap in technological efficacy between the two generations due to the differences between digital immigrants and digital natives.^3^ According to Wang and Cheng (2022), AI-related publications in education are mostly found in China, followed by the US and UK. We hypothesize that Asian researchers are the most open to AI-assisted screening tools.

Q4: Do researchers' patterns of tool usage differ by continents, years of experience in systematic reviews, and age?

H4: Since most tools were developed in the US, Canada, UK, Netherlands, and Germany, researchers from these countries may receive information and training about the software more conveniently from conferences and events compared to researchers who live in other countries. We hypothesize that researchers located in North America and Europe use more tools compared to researchers from other countries. Since researchers with more experience already established a supportive professional network to keep up with the latest updates in the systematic review arena, we hypothesize that researchers with longer years of experience or have conducted more systematic reviews use more tools than researchers with shorter years of experience.

## 5 Method

### 5.1 Study design

This mixed-methods study received approval from the Institutional Review Boards of the authors' affiliated institutions. We collected data using a Qualtrics survey that included both open-ended and closed-ended questions. A codebook of the survey is available in the Supplementary material. Participants began the survey by reviewing the purpose of the survey and confirming their voluntary participation in the research. The main body of the survey had three sections related to handsearching tools, citation chasing tools, and screening tools. Participants were presented with a list of tools (detailed in Table 1) derived from the authors' expertise and existing literature (Harrison et al., 2020; Zhang and Neitzel, 2024). Then participants were asked to identify the tools they were familiar with and then select and evaluate one citation chasing tool and one screening tool they used most frequently. Evaluation questions were adapted from the System Usability Scale (Brooke, 1995). In addition to these close-ended questions, we included two sets of qualitative questions focused on the use of citation chasing tools and screening tools. Participants were asked to write down the reasons for their choice of the most frequently used tool and to identify barriers that prevented them from using other tools. The survey concluded with demographic questions, covering aspects such as the continent where they conduct research, the number of systematic reviews they had previously conducted, their current position (student or faculty), gender, and age. The Qualtrics survey is available upon request.

### 5.2 Sample recruitment

**Figure 1:**
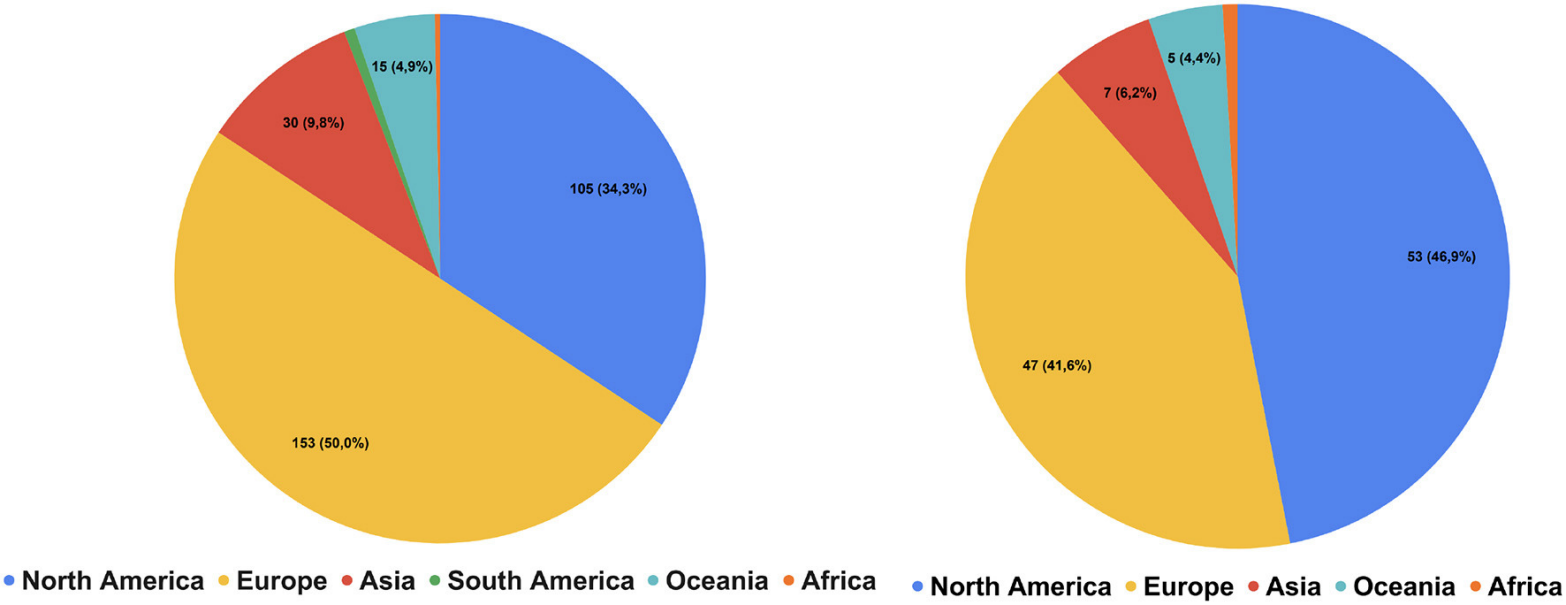
Continents of potential participants reached out through emails **(Left Pie Chart)** and continents of actual participants in survey **(Right Pie Chart)**. In total, we reached out to more than 300 contacts through emails and asked them to share the survey with their colleagues. This does not include participants recruited through social media platforms and institutional email lists. Oceania is mainly represented by Australia.

To be eligible for the survey, participants had to have experience in conducting systematic reviews or meta-analyses. Three strategies were used to recruit participants, adopting a convenience sampling. First, we compiled a list of eligible survey participants. Each author recorded a list of contacts from their research networks. Then we combined this list with the first or the corresponding author of systematic reviews and meta-analyses published in *Review of Educational Research* and *Educational Research Review* in the last 5 years, as well as authors whose contacts were available through educational research centers (Campbell Collaboration, Education Endowment Foundation, What Works Clearinghouse) or authors who have presented at international educational conferences (American Educational Research Association, European Conference of Educational Research, European Association for Research on Learning and Instruction, Hong Kong Education Research Association, Society for Research on Educational Effectiveness). The lists of participants were then combined to delete duplicates. Finally, we sent out emails^4^ inviting these potential participants to take part in our survey and ask them to forward the survey to their colleagues. Figure 1 illustrates the continental distribution of the potential participants we reached out to via email and those who participated in our survey. The left pie chart shows that most of our outreach was directed toward researchers in North America (~50%) and Europe (~41%), with smaller efforts in Asia and Oceania. The right pie chart represents the actual survey participants, mirroring the outreach distribution. This figure highlights the regional focus of our study and underlines the importance of considering geographical biases in tool familiarity and usage. The second strategy was making posts about our survey on Twitter with hashtags to increase exposure of the survey link. The third strategy was asking relevant review institutes or training centers to help distribute the survey through their mailing lists.

### 5.3 Data analysis

We employed quantitative methods to analyze descriptive close-ended questions and qualitative content analysis to delineate open-ended responses. All quantitative analyses were conducted in Stata and R. We report results in means, standards deviations, and percentages. Simultaneously, we present figures for easier interpretations of the descriptive statistics.

Qualitative content analysis (Miles et al., 2014) was used to analyze responses to the open-ended questions. Two questions were asked about both citation chasing tools and screening tools: the reasons for choosing the most commonly used tool and the barriers to using other tools. For each question, we broke down the text into small sentences and assigned labels to these segments based on their meaning. After creating the labels, we iteratively refined our coding to place the responses under the most suitable categories (Forman and Damschroder, 2007; Graneheim et al., 2017). Considering that the types of questions intrinsically contain opposite orders of response (positive reasons vs. negative barriers), the categorization was designed first for positive reasons and then reversed for negative barriers. For example, the categories “easy to use” (positive reason for choosing a certain tool) and “hard to use” (negative barrier preventing respondents to use other tools) share the same topic but opposite formulation. After coding, we grouped the answers by the tool selected and examined the frequencies of each category.

## 6 Results

### 6.1 Sample description

**Figure 2:**
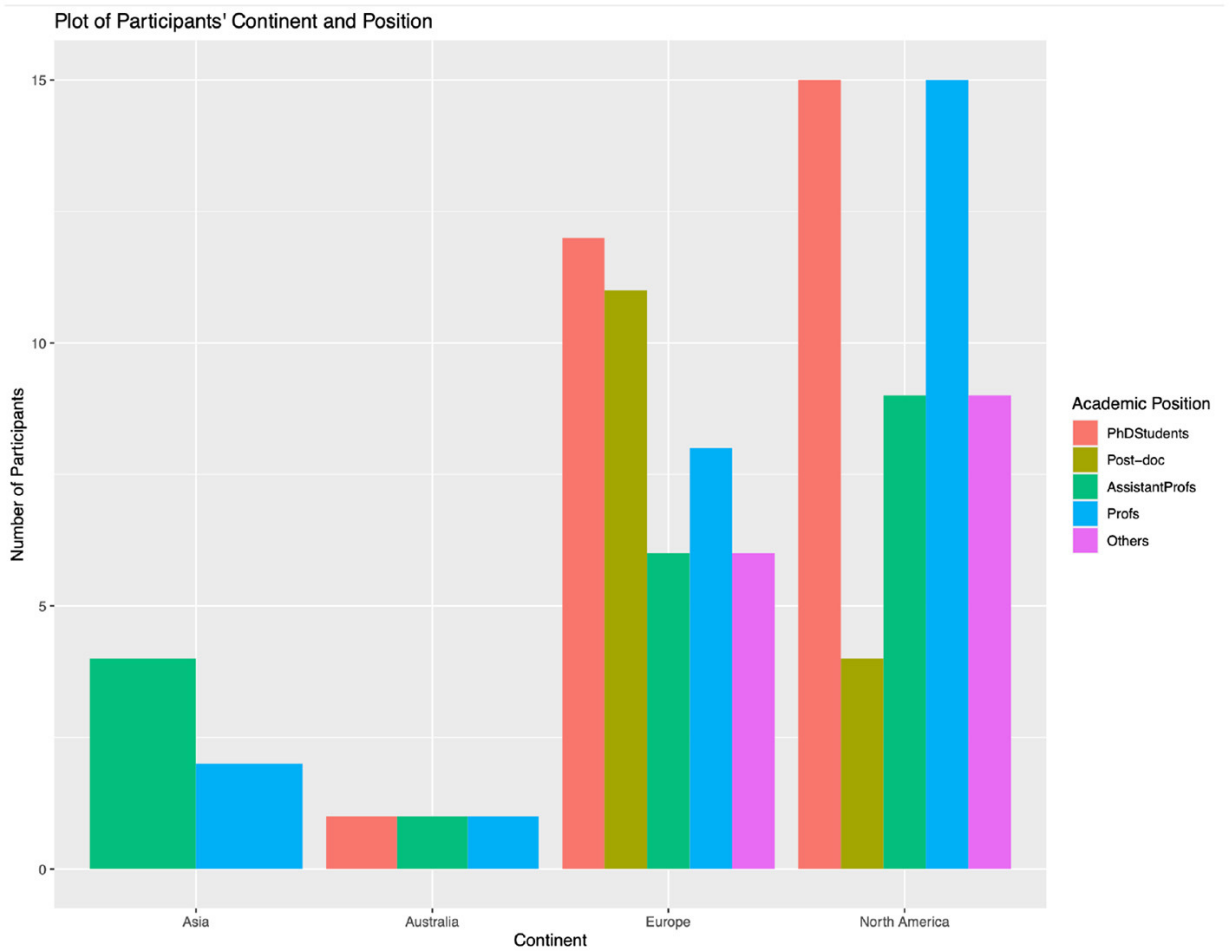
Researchers' academic position distribution by continents.

From June 2022 to January 2023, we collected 218 responses. We removed 34 responses that were completely empty and nine respondents who have never conducted systematic reviews. The final valid sample for analysis had 175 entries. Among these participants, there were slightly more females (57.95%) than males (41.12%). Most participants came from the 25–34 years old age group (39.62%), 35–44 years old age group (37.74%), and 45–64 years old age group (20.75%). In terms of current position, there was diversity in students and faculties, with 17.71% undergraduate or master's students, 14.85% assistant professors, 12.00% post-doctoral researchers, and 8.57% PhD students. As for continents, most participants conduct their research mainly in North America (50.00%) and Europe (41.35%). In terms of number of review projects conducted, most researchers (39.62%) have conducted 1–2 reviews, 33.02% conducted 3–4 reviews, and 27.36% conducted 5 or more. All researchers have conducted systematic review projects before: 18.85% had 0–2 years of experience, 19.42% had 2–4 years of experience, 11.42% had 4–6 years of experience, and 9.71% had 6 or more years of experience. Table 2 shows more details about the participants' demographic information. Figure 2 presents the distribution of the academic positions of researchers across different continents. The data reveal that North America has a more diverse representation of academic positions, including a significant number of doctoral students, assistant professors, and associate/full professors. In contrast, Europe has a higher concentration of mid to senior-level faculty members. This figure emphasizes the potential influence of academic position on the familiarity and use of systematic review tools, which may differ significantly by region.

**Table 2:** Participants' descriptive statistics.

**Variable**	**Category**	***n***	**Percent**	**Cumulative**
Gender	Male	44	41.12	41.12
	Female	62	57.94	99.07
	Other	1	0.93	100
Age	18–24 years old	1	0.94	0.94
	25–34 years old	42	39.62	40.57
	35–44 years old	40	37.74	78.3
	45–64 years old	22	20.75	99.06
	Above 64	1	0.94	100
Current position	Doctoral student	31	28.44	28.44
	Post-doc	15	13.76	42.2
	Faculty (assistant)	21	19.27	61.47
	Faculty (associate or Full)	26	23.85	85.32
	Non-faculty research position	3	2.75	92.66
	Non-university research position	8	7.34	100
	Others^a^	5	4.59	89.91
Continent	North America	52	50.00	50.00
	Asia	6	5.77	55.77
	Europe	43	41.35	97.12
	Oceania/Australia	3	2.88	100
Number of review projects conducted	1–2	42	39.62	39.62
	3–4	35	33.02	72.64
	5 or more	29	27.36	100
Years of experience conducting reviews in education	0–2	33	31.73	31.73
	2–4	34	32.69	64.42
	4–6	20	19.23	83.65
	6 or more	17	16.35	100

^a^Clinical faculty, research assistant, librarian, junior researcher, and educational support.

### 6.2 Handsearching tools

As for current practices conducting handsearching, we hypothesized in Section 4 that researchers either skip this search step or manually conduct handsearching. Consistent with our hypothesis, 61.93% of the respondents manually browse through journals' or conferences' websites, 26.14% reported using certain tools, and 11.93% never conduct handsearching. As for time spent on handsearching, around half of the participating researchers reported spending < 10 h (49.47%), 30.53% reported spending 10–20 h, and 20% reported spending more than 20 h. When we asked participants what tools they use to conduct handsearching, researchers mentioned their university's library tools, journal websites, databases, and looking through journals manually.

### 6.3 Citation chasing tools

#### 6.3.1 Researchers' current choices

**Figure 3:**
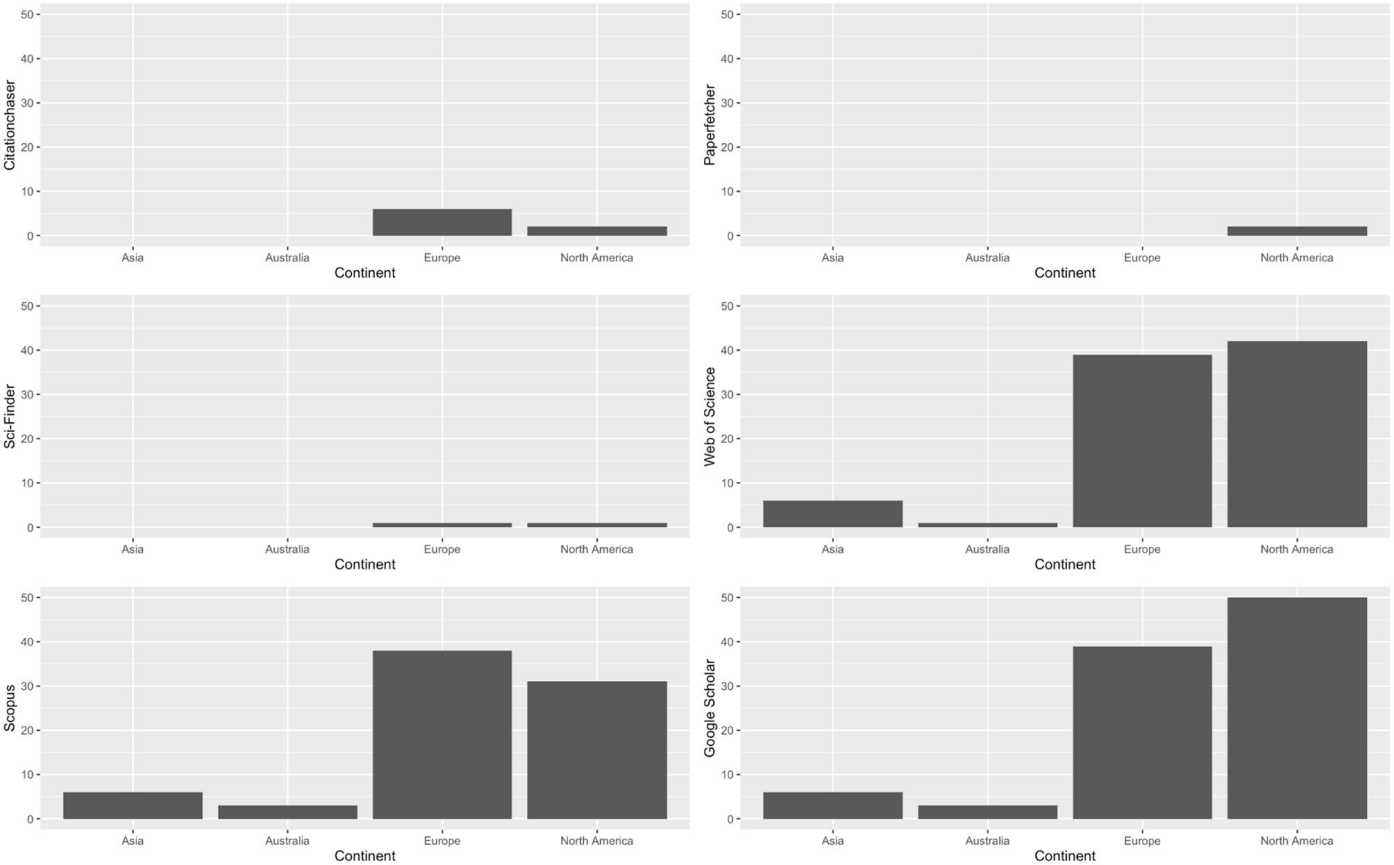
Citation chasing tools that participants have heard of (multiple selections).

**Figure 4:**
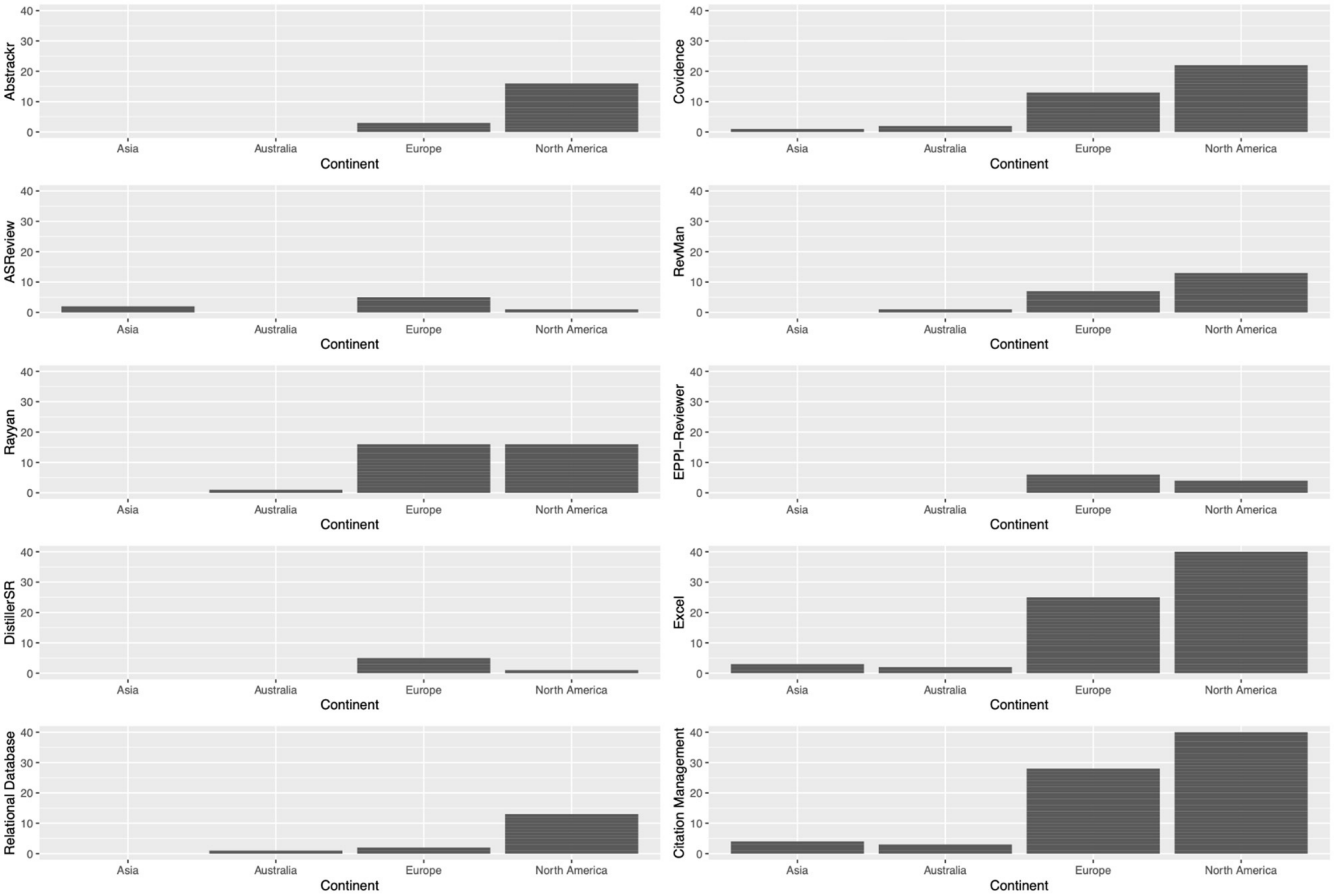
Screening tools that participants have heard of (multiple selections per participant).

We asked researchers to select citation chasing tools they have heard about from a list generated by authors (presented in Table 1). Databases seem to be the most heard about sources, with Google Scholar being mentioned 33.79% of times, Web of Science being mentioned 29.02% of times, and Scopus being mentioned 26.53% of times. Sci-Finder was mentioned 2.72% of times, drawing a tie with Citationchaser. Paperfetcher was mentioned 1.36% times and SpiderCite was mentioned 0.45% times. Figure 3 presents tools participants have heard by breaking down by specific tools and continents where researchers conduct research in. Then we inquired upon the most frequently used tool. Again, the three databases were mentioned the most, with Google Scholar being mentioned 64.05% times, Web of Science being mentioned 14.38% times, and Scopus being mentioned 11.76% times. This figure underscores the regional disparities in tool awareness, which may influence the efficiency and outcomes of systematic reviews conducted in different parts of the world. Figure 4 depicts the most frequently used citation chasing tools among researchers, categorized by continent. The figure highlights several key trends in tool usage across different regions.

Moreover, we asked users to rate the tools based on speed at learning, requirement of technical assistance, complexity, and satisfaction with the tool. The overall satisfaction with Google Scholar is 3.94 (*n* = 90), SpiderCite is 3.61 (*n* = 7), CitationChaser is 3.45 (*n* = 5, *sd* = 0.54), Web of Science is 3.38 (*n* = 20), Scopus is 3.22 (*n* = 16), and Sci-Finder is 2.75 (*n* = 1). Cronbach's alpha for this scale is 0.63.

#### 6.3.2 Reasons and barriers behind tool selection

We asked participants to state the reasons behind choosing their preferred tool. A qualitative analysis of the open-ended responses reveals that most individuals (57.14%) have justified their choice of tool based on ease of use. For respondents, an easy-to-use tool is accessible on the web without the need for downloading, requires minimal time to learn its features, and is fast in executing workflow steps such as logging in and conducting searches. Additionally, it offers relevant functionalities like importing records from databases, supporting simultaneous collaboration, and transferring datasets into statistical software. Examples of quotes include “it just feels easier or more accessible and faster” and “it is handy and easy to use.” The second category is comprehensiveness and functionality (25.89%). Most of the answers highlight that tools are considered functional when they have all the requirements to trace quality and broad literature. Some researchers consider a tool's functionality by whether it is well-accepted among the scientific community. For example, some respondents stated that Scopus “finds the most relevant paper,” “it's well-known and accepted by the scientists of my area,” or about Web of Science that “it is a comprehensive well-indexed tool.” The third category is knowledge and habits (12.5%), which refers to users' familiarity with the tool. Sometimes, users continue to rely on one tool although they know the existence of other tools. This is shown in this example “I am just not familiar with them. I am certain there are better tools and would love to learn much more about them!”

Then we asked participants to state obstacles that stop them from using other tools. The first barrier is the lack of knowledge (38.70%). This category contains both researchers who have never heard of other tools and researchers who said that they are “not familiar with most of these tools.” The second barrier is the lack of comprehensiveness (16.12%). This category contains the irreproducibility of the research, the lack of a way to extract and organize the results easily, and limited research coverage. The third barrier is the high cost of certain tools or the fact that their university does not provide access (15.05%). The fourth obstacle is difficulty of use (12.90%), which refers to different technical aspects. Example quotes include “their interface might need more time and effort to do a search,” “inability to bulk download,” or “difficulty logging in.” Finally, lack of time (10.75%) prevents researchers from using a tool properly. Participants explained that it is necessary to spend a lot of time learning its functionalities and understanding how it works. Some respondents say that “everything takes time to learn. I don't have time,” or “I do not have much time to learn new tools, so I prefer to use the one that I am familiar with.” Others perceive that the time investment required may not lead to a real benefit. This can be shown in this comment: “I would need to invest time to learn how to use them, and the benefits are unclear to me.”

#### 6.3.3 Preferences by continents and age

Researchers from Asia and Australia have only heard about databases, such as Web of Science (*n* = 10), Scopus (*n* = 12), and Google Scholar (*n* = 12). Apart from databases, researchers from Europe have heard about Citationchaser (*n* = 6), Sci-Finder (*n* = 1), and others (e.g., ERIC, EBSCO, and connected papers); researchers from North America have heard about Citationchaser (*n* = 8), Paperfetcher (*n* = 2), Sci-Finder (*n* = 2), and others (e.g., PsycInfo, ERIC, PsysNEY, Connected papers, Research rabbit, refworks, and Scite).

In terms of age, researchers under 24 years old have only heard about databases; researchers above 45 years old have also only heard about databases, with 1 person mentioning Sci-Finder; researchers between 25 and 44 years old are most updated with citation software development, with eight people mentioning Citationchaser, three people mentioning Paperfetcher, two people mentioning Sci-Finder, and eight selecting others (e.g., Eric, EBSCO, PsycNET, connected papers, research rabbit, and refworks). We did not find significant variations in tool usage patterns based on years of experience in systematic reviews.

### 6.4 Screening tools

#### 6.4.1 Researchers' current choices

**Figure 5:**
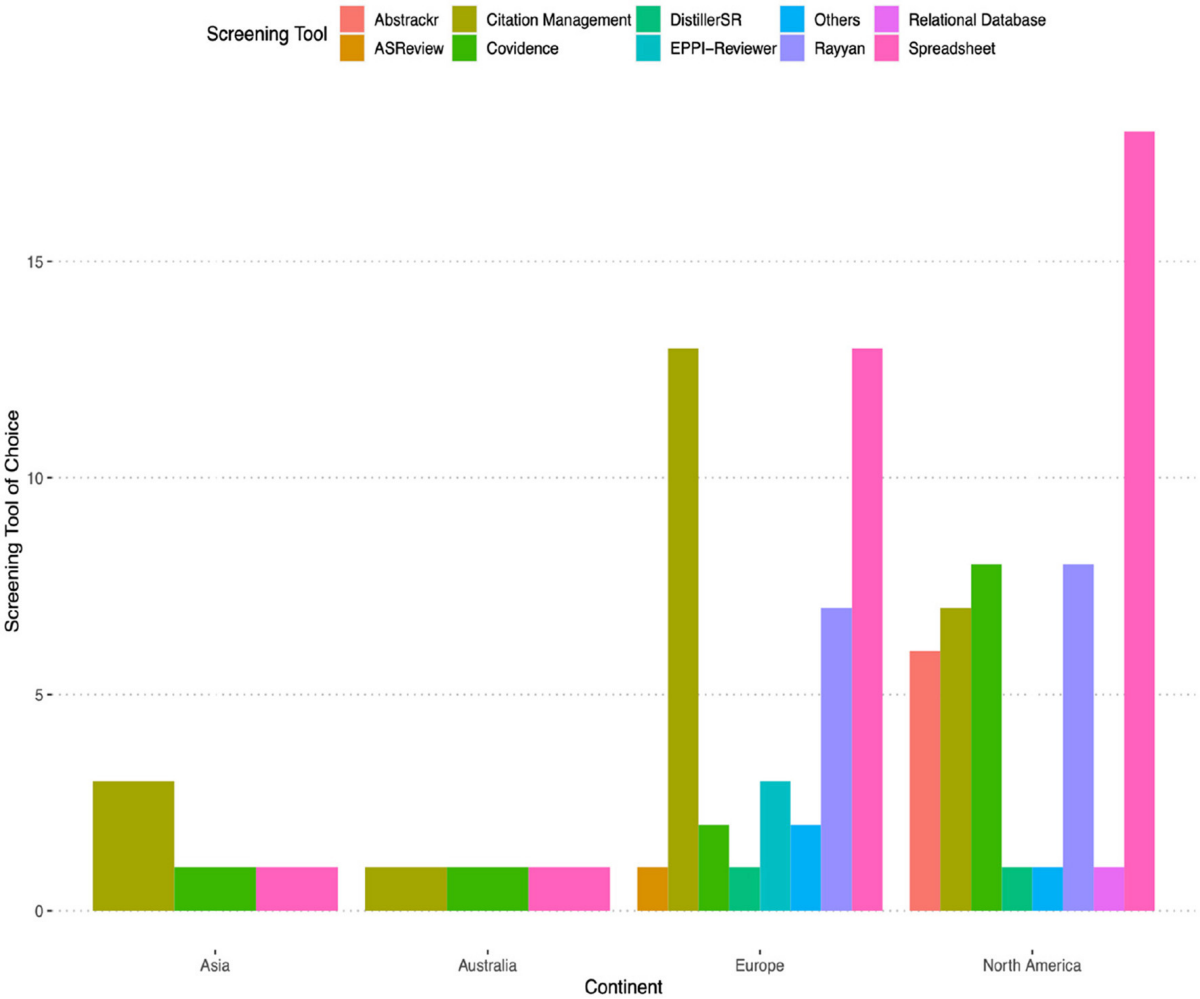
The screening tool each participant has used the most often (one selection per participant).

We surveyed researchers to determine their familiarity with various screening tools. Among the options provided, citation management tools (24.72%) and spreadsheets (24.43%) were the two most commonly recognized screening aids. Researchers also reported knowing Covidence (11.93%), Rayyan (10.23%), RevMan (7.67%), Abstrackr (5.97%), Relational database (4.83%), EPPI-Reviewer (4.26%), ASReview (2.27%), and DistillerSR (2.27%). Figure 5 presents this result with a geographical breakdown.

Next, we investigated the most frequently used tool among the researchers. Once again, spreadsheets (29.37%) and citation management tools (25.40%) emerged as the top two preferred options. They were followed by Rayyan (12.70%), Covidence (11.90%), Abstrackr (5.56%), EPPI-Reviewer (3.97%), ASReview (2.38%), RevMan (1.59%), DistillerSR (1.59%), and relational databases (0.79%).

We asked users to rate the tools based on speed at learning, requirement of technical assistance, complexity, and satisfaction with the tool. The overall satisfaction with ASReview is 4 (*n* = 1), spreadsheet is 3.58 (*n* = 34), Rayyan is 3.5 (*n* = 16), EPPI-Reviewer is 3.5 (*n* = 3), Abtrackr is 3.25 (*n* = 6), Covidence is 3.21 (*n* =14), relational databases is 3 (*n* = 1), citation management software is 2.92 (*n* = 31), DistillerSR is 2.63 (*n* = 2), and RevMan is 2.5 (*n* = 2). Cronbach's alpha for the scale is 0.71.

#### 6.4.2 Reasons and barriers behind tool selection

**Figure 6:**
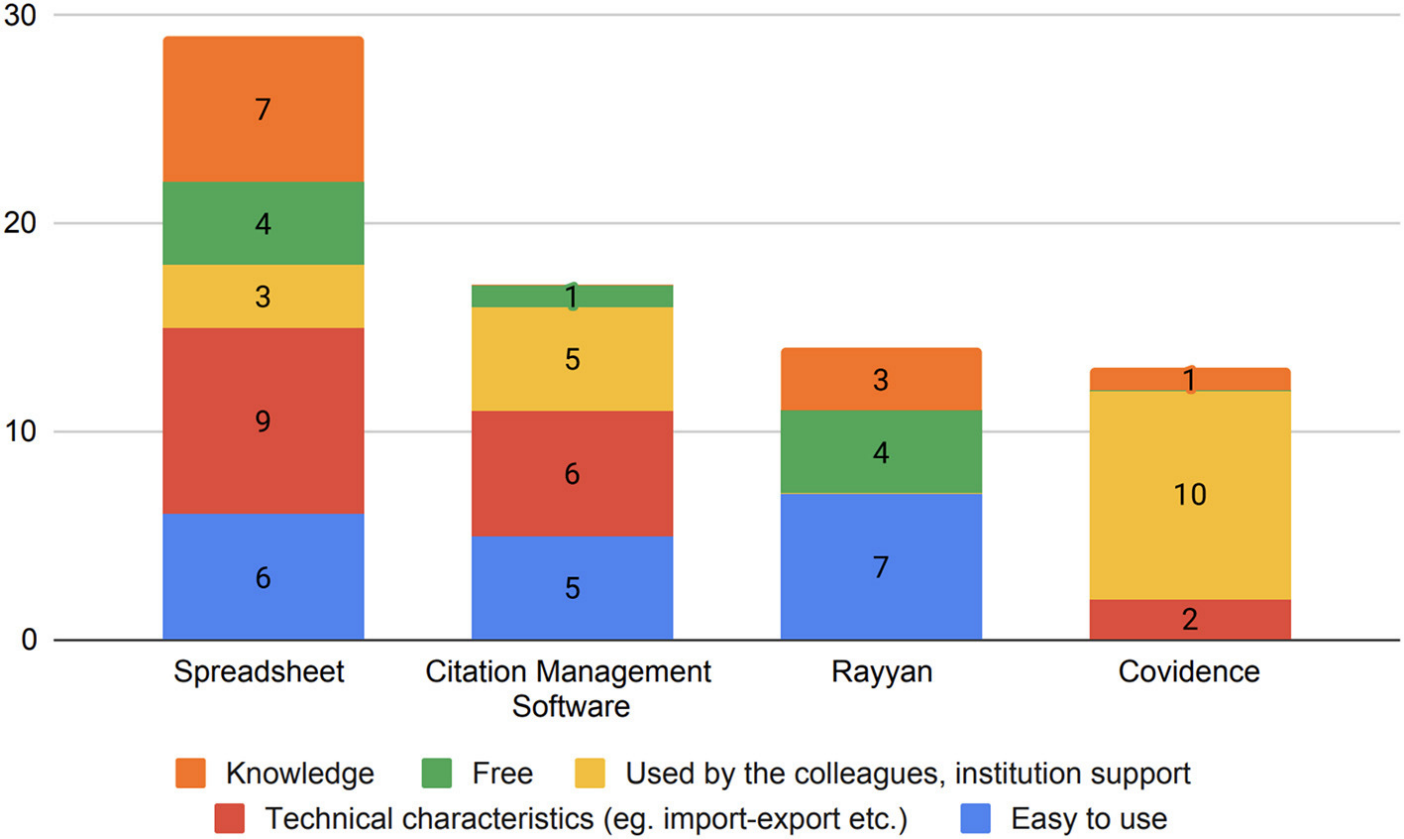
Reasons for researchers to prefer certain screening tools.

Our data reveals that the selection of screening tools is mainly influenced by the scientific environment in which researchers work. Two key factors explain the primary reasons behind tool choices: the support provided by researchers' own institutions and the tool usage patterns among colleagues (19.66%). The second significant factors influencing researchers' tool choices are comprehensiveness and functionality (18.03%). Respondents highlight that “it has a great interface and implements AI to help researchers organize papers. It can speed up the reviewing process,” “I can write down my comments to know the reasons,” and “It has very good features for extracting the data from the articles, helps with inter-rater reliability, and most parameters can be adjusted.” Another significant aspect is the ease of use (17.21%), which encompasses both technical functionality and the initial learning curve. One important technical aspect that contributes to the ease of use of the selected tool is the method of sharing work. Here are a few representative responses we received: “I thought it was the easiest to use and the functionality was appropriate for a systematic review,” “easy to use and easy to collaborate,” “good, reliable documentation, easy to use and easy to share (with other screeners).” Eighteen respondents (14.75%) highlighted that their relationship with a specific screening tool is stable because it is the one they know best. Some respondents highlight that familiarity with the tool is linked to past learning that has not been updated. This is shown in these examples “Honestly, this was how I was trained,” “The first one I used,” “Only one available to me at the time.” The cost factor (9.01%) is another crucial aspect to consider, particularly due to the fact that screening software often incurs initial expenses and ongoing costs for researcher training and software updates. One researcher mentioned that the selected tool is the “cheapest and easiest to maintain without training.” Figure 6 presents reasons behind tool choices.

**Figure 7:**
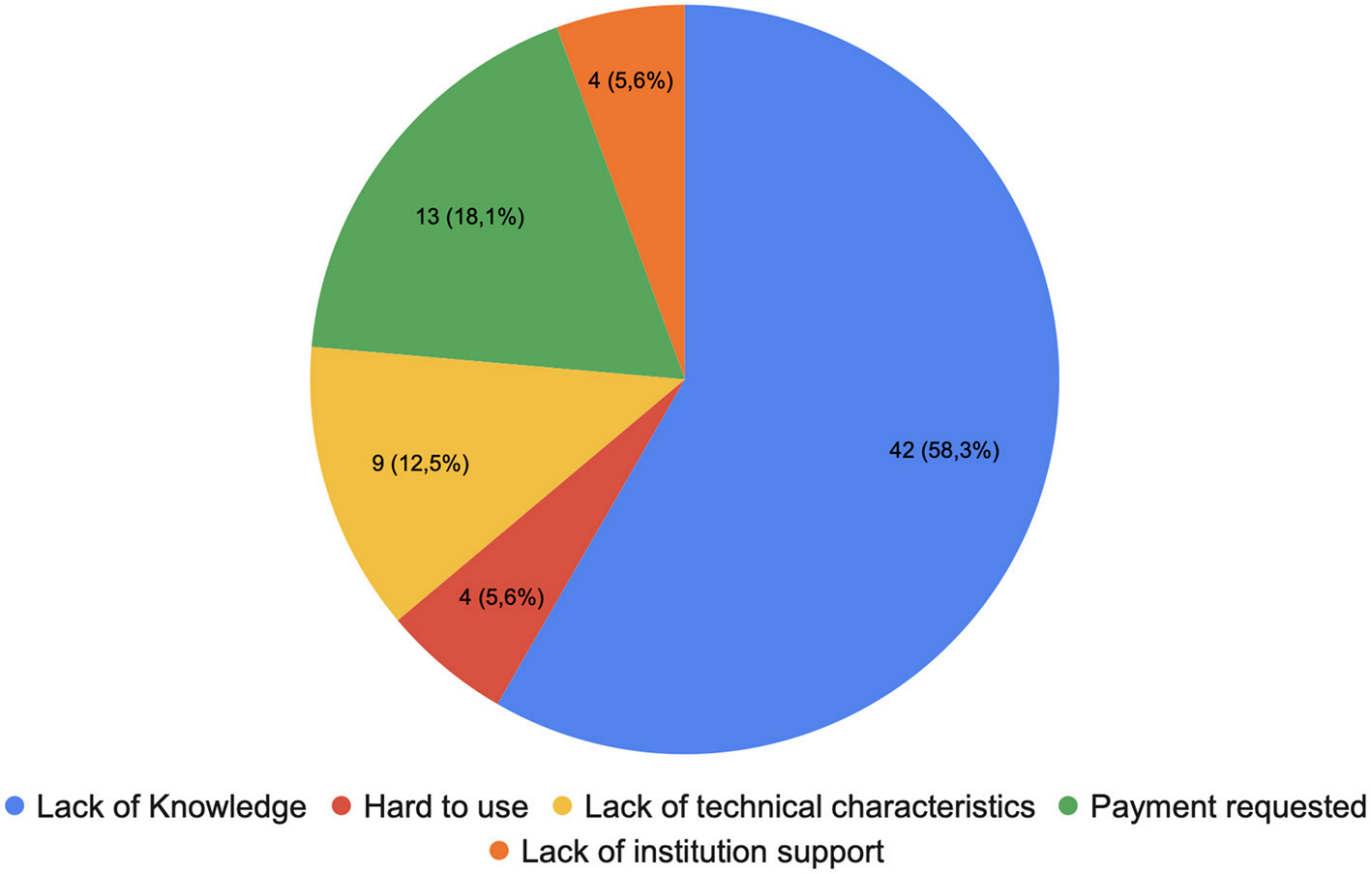
Barriers preventing researchers from using other screening tools.

Then we analyzed the barriers preventing researchers from using other screening tools. The first reason is the lack of knowledge (48.24%) about the tools themselves. The second reason is the cost barrier (18.82%), particularly for individuals who lack university support. Figure 7 presents a visualization of the barriers. Certain screening tools, such as Covidence, which are widely utilized, require the purchase of a license, posing financial challenges for users. One example is: “Covidence charges and it's hard to go back when screening if you mis clicked. Spreadsheets get messy, but I use them for full study screening and coding.” The third reason is dissatisfaction with the functionality and technical comprehensiveness of screening tools (11.76%). For example, they mentioned “less ergonomic,” “time consuming,” and “the other tools are one-off tools, only used for one-step in the process; our team strongly prefers using a tool that can be used for each step in the data collection process. These other tools also aren't ideal for large teams that need to be working on the same project interactively at the same time.”

#### 6.4.3 Preferences by continents and age

When we asked participants whether their review teams are currently using AI-automated functions in systematic reviews, the majority responded “No,” with 39.47% saying “no plan to use at all,” 22.81% “no but plan to use in the future,” and 21.05% “not sure.” Only 16.67% said they are currently using AI-related functions. Among those who reported using AI-assisted tools, 33.33% are using Abstrackr, 22.22% Rayyan, 22.22% other tools (R studio, R packages, or spreadsheets), 11.11% ASReview, and 11.11% DistillerSR.

We then asked participants' attitude toward AI-facilitated tools, with 0 score meaning no plan at all and 3 meaning they are currently using these tools. Contrary to our hypothesis, the group that is the most open to AI tools are assistant professors (*mean* = 1.57), followed by non-university researchers (*mean* = 1.37), followed by associate or full professors (*mean* = 1.16). The group that is the least open to AI tools are post-doctoral researchers (*mean* = 0.93), followed by non-faculty researchers (*mean* = 1), followed by doctoral students (*mean* = 1.07). In terms of continent, most researchers from Asia said “no but plan to use in the future” and most researchers from North America and Europe said “no plan to use at all.” This is consistent with our hypothesis that Asian researchers are more open to AI-assisted tools.

Researchers from Asia have heard about least number of tools, with 2 mentioning Covidence, 2 mentioning ASReview, 1 mentioning RevMan; researchers from Australia mentioned Covidence (*n* = 3), RevMan (*n* = 2), relational database (*n* = 2), and Rayyan (*n* = 1); researchers from North America have heard about Covidence (*n* = 22), Abstrackr (*n* = 16), Rayyan (n = 16), RevMan (n = 13), relational databases (n = 13), EPPI-Reviewer (n = 4), DistillerSR (n = 1), and ASReview (*n* = 1); researchers from Europe have heard about the most number of tools, they mentioned Covidence (n = 35), Rayyan (n = 32), Abstrackr (n = 20), RevMan (n = 20), relational databases (n = 15), EPPI-Reviewer (*n* = 11), ASReview (n = 6), DistillerSR (n = 6), and others: SyRF (*n* = 1), metagear R package (n = 1).

In terms of age, researchers between 25 and 44 years old are most updated with screening tool development since they have heard about all the listed tools and one person even mentioned metagear R package in the others category. We did not find significant variations in tool usage patterns based on years of experience in systematic reviews.

### 6.5 Exploratory analysis

Apart from the research questions, we asked some extra questions for exploratory analysis. We asked the participants to rank the importance of searching strategies. Researchers rank database search as the most important (*mean* = 1.28), handsearching as the least important (*mean* = 3.19), forward (*mean* = 2.77), and backward (*mean* = 2.76) citation chasing as mildly and equally important. In addition to the value assigned to each searching strategy, we also asked for ranking on the time-consuming aspect. Researchers rank handsearching (*mean* = 1.93) as the most time-consuming, database search as the least time-consuming (*mean* = 2.59), forward (*mean* = 2.74), and backward (*mean* = 2.75) as mildly and equally time-consuming.

Furthermore, we asked the participants to rank the importance of screening tools' features. Researchers rank collaboration as the most important (*mean* = 3.60), bulk application (*mean* = 4.15), and accessibility/cost (*mean* = 4.19) as the second most important, followed by deduplication (*mean* = 5.00), blind review (*mean* = 5.21), inter-rater reliability reporting (*mean* = 5.40), process documentation (PRISMA diagram; *mean* = 5.40), research update (*mean* = 5.83), and machine learning (*mean* = 6.11).

## 7 Discussion

In this research, we examined the educational systematic reviewers' tool usage patterns. Consistent with our hypotheses, we found that a majority of researchers still rely on manual handsearching without adopting any tools. One possible explanation is that many researchers are reluctant to adopt new practices because manual handsearching is a familiar and well-understood process. They probably have developed a certain level of comfort and experience with handsearching, making it really difficult to change. Another reason could be limited awareness. The first tool to semi-automate handsearching, Paperfetcher, was developed in 2022. It is likely that researchers need more time to become acquainted with and train the team to adopt new practices. Finally, even when new tools are developed, the sharp learning curve poses challenges. Researchers might find these new tools complex and time-consuming to learn, especially if they are already comfortable with manual searching. Regarding citation chasing tools, we found that databases remain the most popular among researchers, likely because they have been in existence for a longer time. Tools specifically designed for citation chasing purposes seem to be relatively unknown, possibly due to their recent introduction. When it comes to screening tools, it is worth noting that citation management tools and spreadsheets are still the preferred options among researchers, despite not being originally designed for screening purposes. However, among dedicated screening tools, Covidence and Rayyan appear to have a larger user base compared to other tools.

In our investigation into the factors influencing researchers' choices of screening tools, three primary reasons were reported: the research environment (such as institutional support or colleague preferences), the functions of the tools themselves, and ease of use. Several possible explanations can shed light on the reported findings. Regarding the research environment, institutional support and the influence of colleagues play a significant role in shaping researchers' tool preferences. Institutions may provide licenses or resources for specific screening tools, leading researchers to choose those options. Additionally, recommendations from colleagues who have had positive experiences with certain tools can heavily influence researchers' choices. The second factor that emerged from our findings is the functions of the tools. Different screening tools offer various functionalities and features that cater to the specific needs of researchers. Some tools may have advanced search capabilities, AI-automated screening options, or integration with coding tools. Researchers consider these functionalities and assess how well they align with their research requirements and objectives. The functions offered by screening tools play a key role in researchers' decision-making process. Lastly, ease of use was identified as another significant factor influencing researchers' choices. Researchers often prioritize tools that are user-friendly, intuitive, and require minimal technical expertise. The learning curve associated with new tools and the time required to adapt to unfamiliar interfaces can pose challenges. Therefore, researchers tend to opt for tools that are easy to use and integrate seamlessly into their existing workflows.

When exploring the barriers preventing researchers from utilizing alternative tools, the top three reasons identified were a lack of awareness about other tools, dissatisfaction with existing tools, and the high cost associated with alternative options. A lack of knowledge about alternative options can restrict researchers' choices. It is possible that some researchers are unaware of the existence or availability of other tools that could potentially better suit their needs. Dissatisfaction with existing tools suggests that researchers may have encountered limitations or shortcomings with their current choices, leading them to seek alternative solutions. The consideration of cost is crucial, particularly for researchers who lack financial support or have limited budgets, as it affects their ability to acquire and sustain the use of screening tools.

Contrary to our hypothesis, we found that faculties are more open to AI compared to postdoc and doctoral students. There are two possible explanations: the differences in exposure and experience, and the differences in autonomy and freedom. Faculties, being established researchers and educators, have likely witnessed the increasing integration of AI into various domains and have firsthand knowledge of its potential benefits. They may have encountered AI-driven research tools, applications, or publications that have demonstrated the value of AI in their specific areas of expertise. On the other hand, post-doc and doctoral students, although they are highly engaged in research, may still be in the process of gaining familiarity with AI and its applications. They might have less exposure to AI-related advancements or limited opportunities to explore its potential in their research projects. Consequently, their skepticism or hesitation toward AI could stem from a lack of practical experience or a need for further exposure and training. It is also possible that faculties, due to their senior positions, have more decision-making power and autonomy in choosing research tools and methodologies. This increased authority may make them more open to exploring and embracing innovative technologies like AI, whereas post-doc and doctoral students might have less influence or freedom to adopt new approaches.

Furthermore, when examining the data by continent, we observed that researchers from Europe had greater awareness of screening tools compared to researchers from North America, Australia, and Asia. However, since we only managed to obtain six responses from Asia, three responses from Australia, and researchers working in North America might originally come from Asia or other continents, findings related to continents should be interpreted with caution. Notably, the tool *Citationchaser* had the highest recognition in Europe, while *Paperfetcher* was most commonly known among researchers in North America. This discrepancy aligns with the fact that *Citationchaser* was developed in the Netherlands, and *Paperfetcher* originated in the United States. In terms of popularity within specific regions, the three most widely used screening tools in North America were Covidence, Rayyan, and Abstrackr. Meanwhile, in Europe, the top three screening tools were Rayyan, EPPI-Reviewer, and Covidence. It is worth noting that many of the commonly used tools were developed in the United States, Canada, the United Kingdom, the Netherlands, and Germany. Researchers from these countries may have an advantage in terms of accessibility to information and training through conferences and events that focus on these tools. This facilitates knowledge sharing, recommendations, and training opportunities, ultimately leading to increased awareness and utilization of these tools. Conferences and events held in countries where tools originate may serve as important platforms for showcasing and promoting these tools. Researchers attending these events are more likely to be exposed to the latest advancements, receive training, and engage in discussions related to the usage of these tools. Institutions located in countries with higher tool awareness often provide institutional support, including funding and resources dedicated to software training. They may prioritize offering training programs, workshops, and support to encourage researchers to adopt and effectively utilize these tools. The distribution of tool awareness and popularity across different continents and regions highlights how the origin, development, and accessibility of tools influence researchers' knowledge and adoption.

### 7.1 Implications for reviewers and tool developers

The findings of this study have significant implications for researchers and tool developers alike. A key finding indicates that many researchers are not utilizing available tools due to a lack of awareness about their existence. It is important to recognize that systematic reviewers fall into two groups: those specializing in research synthesis, for whom staying up to date with tools is part of their expertise, and those conducting systematic reviews as part of a broader research project, where this may be the only large review they conduct. Raising awareness among the former group is typically easier, as they naturally follow methodological developments. However, for researchers who do not regularly conduct systematic reviews, the lack of awareness stems from limited exposure to such tools. Therefore, outreach efforts must be tailored to both groups: simplifying tool access and training for those who need it infrequently and offering continuous professional development for specialists. Tool developers can play a proactive role in facilitating tool adoption by offering professional development sessions in both asynchronous and synchronous formats. These sessions can help researchers gain proficiency in using new tools and adapt to their functionalities, thus enabling a smoother integration of innovative tools into research workflows.

To address this, it is crucial to foster discussions about newly developed tools on global platforms to increase researchers' awareness of the available options. This awareness-building effort should particularly target researchers in Asia and Australia, as most tools have not been developed in those regions. Additionally, libraries and information retrieval specialists can play a crucial role as intermediaries. University libraries, often serving as resource hubs for systematic reviews, can be powerful partners in disseminating information and training researchers in the use of systematic review tools. Engaging with librarians or information retrieval specialists to promote these tools will foster a shift in the institutional environment, ensuring better access and support for faculty members conducting reviews.

Another factor contributing to the underutilization of tools is the absence of transparent reporting on tool usage in publications (Zhang and Neitzel, 2024). Researchers should strive to adopt open science practices, not only by openly reporting which tools they use but by sharing detailed documentation of the review process itself. This is critical for improving transparency and reproducibility, particularly as education research moves toward more open and accessible standards. Tools designed specifically for systematic reviews offer an advantage over traditional methods like Excel spreadsheets by enabling researchers to track and document decisions in a way that is both transparent and reproducible. In this context, tool adoption is not just about efficiency—it is essential for producing high-quality, reproducible research that aligns with open science principles.

In addition, researchers have expressed a preference for tools that can handle various stages of the review process, including screening, full-text review, and coding. However, they have also noted limitations in the comprehensiveness of existing tools. To address this, systematic tool developers should prioritize conducting user experience research to gain a better understanding of both novice and expert users' needs. This understanding can inform an iterative product development approach, leading to improvements in tools and making them more effective and user-friendly. Furthermore, a lack of knowledge has emerged as the most significant barrier to adopting new tools. The increasing number of tools available for systematic reviews has made it challenging for researchers to choose the one that best fits their needs. One strategy to overcome this barrier is to provide resources on the tool's website, such as videos and instructional materials, that demonstrate its functionalities. Another strategy could be to incorporate information about new tools into existing training programs for conducting systematic reviews.

Cost has also emerged as a significant barrier to tool usage. For example, the survey participants highlighted the popularity of Rayyan; however, its recent transition to charge higher prices might impact its user groups. In light of this, it is crucial for tool developers to consider the affordability of their tools to ensure wider accessibility.

Finally, the growing demand for timely evidence in the field of education is also driving the need for more efficient research synthesis methods. In addition to traditional systematic reviews and meta-analyses, there is increasing interest in alternative forms of research synthesis, such as scoping reviews, evidence gap maps, and living reviews. These types of reviews often require tools that support ongoing updates and faster data processing. As new methodologies emerge, systematic review tools will need to evolve to accommodate these shifts, providing the necessary speed and efficiency to meet the demands of the field.

### 7.2 Limitations and directions for future research

It is important to acknowledge several limitations when interpreting the results of this study, and future research could extend our understanding of these findings. Firstly, this research solely focused on the users' perspectives regarding tools for searching and screening studies. However, many tools now facilitate multiple stages of the review process, including data extraction, analysis, and reporting, functions that were not investigated in this study. Some participants noted the potential benefits of platforms that support multiple stages of the review process, providing added convenience and integration. Future research should explore the use of comprehensive tools that span multiple stages of systematic reviews to assess their usability, efficiency, and potential for improving the overall review process. Additionally, future studies could investigate whether tool usage varies by type of review methodology, such as traditional systematic reviews, scoping reviews, or living reviews, as each type may have different needs and demands for tool functionality.

Secondly, it is important to note that our participant sample size was relatively small, consisting of only 175 participants. We used a convenience sampling method, which carries inherent limitations and poses potential threats to the external validity of the results. Therefore, caution should be exercised when generalizing these findings to the entire population of systematic reviewers in the field of education. Furthermore, it is worth mentioning that most of the researchers we contacted for participation were from research institutions in North America and Europe, which aligns with the geographic location of the authors conducting this study. This recruitment strategy may have introduced selection bias and limited the representation of researchers from other continents or regions. In addition, we encountered a significant amount of missing data for the open-ended questions. After identifying the most frequently used citation chasing and screening tools, a considerable portion of participants (44–58%) did not provide reasons for their tool selection or barriers preventing them from using other tools. Consequently, some of the conclusions drawn from this study should be interpreted as specific to the subset of participants who responded to these open-ended questions. Given these limitations, further research with larger and more diverse samples, employing probability sampling methods, and addressing the issue of missing data would help enhance the generalizability and reliability of the findings.

Thirdly, it is important to acknowledge that this study did not assess the actual usability and performance of the tools under investigation. While we collected users' opinions and preferences through the survey, we did not conduct in-depth evaluations of the tools' usability. Future investigations could consider incorporating methods used by Harrison et al. (2020) in their study on reviewers in healthcare, such as asking users to engage in trial projects using selected software tools and report their firsthand experiences. Additionally, studies should move beyond usability testing to focus on quantifying the efficiency and resource savings these tools offer. Documenting metrics like time saved, reductions in required personnel, and workflow improvements would provide the kind of hard data that can convince more researchers to adopt these tools. These comparisons could provide valuable insights into the practical benefits of tools like ASReview, Covidence, and Rayyan.

Additionally, future studies should explore whether systematic reviews that use these tools produce more accurate and reliable results compared to those that rely on traditional, manual methods. Research could examine whether studies using advanced systematic review tools yield different conclusions, smaller effect sizes, or fewer errors, as has been done with studies investigating the impact of open science practices on research outcomes. Such studies would provide evidence of whether using these tools improves not only efficiency but also the overall quality and trustworthiness of systematic reviews.

Our findings on attitudes toward AI-assisted tools revealed unexpected results, with faculty members being more open to using AI tools than post-docs and doctoral students. This highlights the importance of understanding how career stage, experience, and exposure to new technologies influence attitudes toward AI-driven tools. Future research could explore these dynamics further by conducting interviews with researchers at different career stages, seeking to understand their motivations, hesitations, and experiences with AI-assisted tools. Additionally, investigating whether AI-assisted software provides differential support across career stages and research fields could uncover important insights for tool developers and research institutions.

Given the growing interest in AI-based tools for screening and data extraction, future studies should also compare the performance of various AI-assisted tools (e.g., ASReview, Covidence, and Rayyan) in education-related systematic reviews. Retrospective analyses of previously conducted reviews, screened using both manual and AI-assisted methods, would provide valuable data on the proportion of studies missed, time saved, and overall efficiency. While similar evaluations have been conducted in healthcare (e.g., Chai et al., 2021; Gates et al., 2019; Olofsson et al., 2017), few studies have examined these tools in the context of educational research. Given the increasing demand for timely evidence in education, such evaluations could help researchers select the most appropriate tools to conduct efficient, high-quality reviews.

Longitudinal studies are also needed to monitor how tool adoption evolves over time, particularly as AI and other advanced technologies become more integrated into the review process. Tracking shifts in tool usage and researcher preferences over extended periods will provide deeper insights into the factors influencing long-term adoption. This can also provide valuable data on how external factors, such as the release of new tools, pricing changes, or institutional support, impact tool uptake and sustained use.

Finally, frameworks like the Diffusion of Innovations (Rogers, 2003) could offer valuable insights into the adoption patterns of systematic review tools. By examining how different segments of the research community adopt these tools—whether they are innovators, early adopters, or part of the majority—researchers can develop strategies to increase the diffusion of new tools. Such studies could explore which factors (e.g., ease of use, cost, peer influence, and institutional support) most influence adoption at various stages. This approach could provide actionable guidance on how to promote widespread adoption, ensuring that new tools gain traction across the full spectrum of researchers, from early-career scholars to experienced professionals.

## 8 Conclusion

This study provides the first comprehensive survey on the current usage of tools by educational systematic reviewers, with a unique focus on participants from different countries, enabling a cross-continental comparison of tool usage trends. These findings help identify regions that are more or less aligned with the latest developments in systematic review tools, providing valuable insights into global patterns. The results show that, compared to fields like biomedical and healthcare research, educational researchers lag behind in adopting purpose-built tools, with many still relying on spreadsheets. In contrast, tools like Abstrackr, Rayyan, and Covidence have gained greater traction in other disciplines (Harrison et al., 2020).

However, this study also highlights the substantial room for improvement in the educational systematic review process, which is increasingly driven by the need for both efficiency and accuracy. By bringing attention to the benefits of these tools—not only in terms of time savings and streamlined workflows but also in improving transparency and reproducibility—the study encourages broader tool adoption across the education field. As education research moves toward more open science practices, the ability to document and share systematic review processes in a transparent, reproducible manner becomes critical. Tools designed for systematic reviews are essential for achieving these goals, moving beyond manual processes that limit transparency.

Looking forward, future research should explore critical areas such as usability testing, documenting efficiency gains, and evaluating how these tools impact the accuracy and reliability of reviews compared to traditional methods. The use of AI-based tools for screening and data extraction, in particular, shows promise for reducing human error and expediting the review process, warranting further investigation. Additionally, applying frameworks like the Diffusion of Innovations theory could provide valuable insights into how new tools spread across the research community, offering guidance on how to increase adoption among researchers with varying levels of expertise.

By shedding light on the current state of tool usage and providing directions for future research, this study aims to promote a more systematic, transparent, and efficient approach to conducting educational systematic reviews. Encouraging the adoption of these tools will be key to meeting the growing demand for timely, reliable evidence in education research.

## Data Availability

The raw data supporting the conclusions of this article will be made available by the authors, without undue reservation.

## Footnotes

^1^Transparency refers to explicitly detailing the elements of the review process, reproducibility refers to the possibility for a third party to replicate the review process (Cram et al., 2020).

^2^This study was preregistered at Open Science Framework (https://osf.io/4y5gn/) on October 18th, 2022.

^3^Today's young people have been called “digital natives” in past literature, which differentiates them from the previous generation of “digital immigrants” (Prensky, 2001).

^4^Email invitation template is available in Supplementary material. A complete list of educational systematic reviewers' emails and names is available upon request.

## References

[B1] Alexander P. A. (2020). Methodological guidance paper: the art and science of quality systematic reviews. Rev. Educ. Res. 90, 6–23. 10.3102/003465431985435238293548

[B2] Blaizot A. Veettil S. K. Saidoung P. Moreno-Garcia C. F. Wiratunga N. Aceves-Martins M.. (2022). Using artificial intelligence methods for systematic review in health sciences: a systematic review. Res. Synth. Methods 13, 353–362. 10.1002/jrsm.1553 35174972

[B3] Borah R. Brown A. W. Capers P. L. Kaiser K. A. (2017). Analysis of the time and workers needed to conduct systematic reviews of medical interventions using data from the PROSPERO registry. Br. Med. J. Open 7:e012545. 10.1136/bmjopen-2016-012545 28242767 PMC5337708

[B4] Brooke J. (1995). SUS-A quick and dirty usability scale. Usabil. Eval. Indus. 189, 4–7.

[B5] Carey N. Harte M. Mc Cullagh L. (2022). A text-mining tool generated title-abstract screening workload savings: performance evaluation versus single-human screening. J. Clin. Epidemiol. 149, 53–59. 10.1016/j.jclinepi.2022.05.017 35654270

[B6] Chai K. E. Lines R. L. Gucciardi D. F. Ng L. (2021). Research Screener: a machine learning tool to semi-automate abstract screening for systematic reviews. Systemat. Rev. 10, 1–13. 10.1186/s13643-021-01635-3 33795003 PMC8017894

[B7] Clark J. Glasziou P. Del Mar C. Bannach-Brown A. Stehlik P. Scott A. M. (2020). A full systematic review was completed in 2 weeks using automation tools: a case study. J. Clin. Epidemiol. 121, 81–90. 10.1016/j.jclinepi.2020.01.008 32004673

[B8] Cooper C. Booth A. Varley-Campbell J. Britten N. Garside R. (2018). Defining the process to literature searching in systematic reviews: a literature review of guidance and supporting studies. BMC Med. Res. Methodol. 18:85. 10.1186/s12874-018-0545-3 30107788 PMC6092796

[B9] Cooper H. Hedges L. V. Valentine J. C. (2019). The Handbook of Research Synthesis and Meta-analysis. New York, NY: Russell Sage Foundation.

[B10] Cram W. A. Templier M. Paré G. (2020). (Re) considering the concept of literature review reproducibility. J. Assoc. Inform. Syst. 21, 1103–1114. 10.17705/1jais.00630

[B11] Davies P. (2000). The relevance of systematic reviews to educational policy and practice. Oxf. Rev. Educ. 26, 365–378. 10.1080/713688543

[B12] Forman J. Damschroder L. (2007). “Qualitative content analysis,” in Empirical Methods for Bioethics: A Primer, Vol. 11, eds. L. Jacoby and L. A. Siminoff (Bingley: Emerald Group Publishing Limited), 39–62.

[B13] Gates A. Guitard S. Pillay J. Elliott S. A. Dyson M. P. Newton A. S.. (2019). Performance and usability of machine learning for screening in systematic reviews: a comparative evaluation of three tools. Systemat. Rev. 8, 1–11. 10.1186/s13643-019-1222-2 31727150 PMC6857345

[B14] Graneheim U. Lindgren B. Lundman B. (2017). Methodological challenges in qualitative content analysis: a discussion paper. Nur. Educ. Tod. 56, 29–34. 10.1016/j.nedt.2017.06.002 28651100

[B15] Haddaway N. Grainger M. Gray C. (2022). Citationchaser: a tool for transparent and efficient forward and backward citation chasing in systematic searching. Res. Synth. Methods 13:1563. 10.1002/jrsm.1563 35472127

[B16] Harrison H. Griffin S. J. Kuhn I. Usher-Smith J. A. (2020). Software tools to support title and abstract screening for systematic reviews in healthcare: an evaluation. BMC Med. Res. Methodol. 20:7. 10.1186/s12874-020-0897-3 31931747 PMC6958795

[B17] Institute for Evidence-Based Healthcare (2021). SR-Accelerator. Systematic Review Accelerator. Available at: https://sr-accelerator.com/#/help/spidercite (accessed October 18, 2023).

[B18] Janssens A. C. J. W. Gwinn M. (2015). Novel citation-based search method for scientific literature: application to meta-analyses. BMC Med. Res. Methodol. 15:84. 10.1186/s12874-015-0077-z 26462491 PMC4604708

[B19] Lefebvre C. Glanville J. Briscoe S. Littlewood A. Marshall C. Metzendorf M. I.. (2022). “Searching for and selecting studies,” in Cochrane Handbook for Systematic Reviews of Interventions Version 6.3, eds. J. P. T. Higgins, J. Thomas, J. Chandler, M. Cumpston, T. Li, M. J. Page, et al. (Cochrane), 67–107. Available at: www.training.cochrane.org/handbook (accessed October 18, 2023).

[B20] Miles M. B. Huberman A. M. Saldaña J. (2014). Qualitative Data Analysis: A Methods Sourcebook. Thousand Oaks, CA: Sage Publications.

[B21] Ming N. C. Goldenberg L. B. (2021). Research worth using: (re) framing research evidence quality for educational policymaking and practice. Rev. Res. Educ. 45, 129–169. 10.3102/0091732X2199062038293548

[B22] Olofsson H. Brolund A. Hellberg C. Silverstein R. Stenström K. Österberg M.. (2017). Can abstract screening workload be reduced using text mining? user experiences of the tool Rayyan. Res. Synth. Methods 8, 275–280. 10.1002/jrsm.1237 28374510

[B23] Ouzzani M. Hammady H. Fedorowicz Z. Elmagarmid A. (2016). Rayyan-A web and mobile app for systematic reviews. Systemat. Rev. 5:210. 10.1186/s13643-016-0384-4 27919275 PMC5139140

[B24] Page M. J. McKenzie J. E. Bossuyt P. M. Boutron I. Hoffmann T. C. Mulrow C. D.. (2021). The PRISMA 2020 statement: an updated guideline for reporting systematic reviews. Systemat. Rev. 10, 1–11. 10.1186/s13643-021-01626-4 33781348 PMC8008539

[B25] Pallath A. Zhang Q. (2023). Paperfetcher: a tool to automate handsearching and citation searching for systematic reviews. Res. Synth. Methods 14, 323–335. 10.1002/jrsm.1604 36260090

[B26] Pigott T. D. Polanin J. R. (2020). Methodological guidance paper: High-quality meta-analysis in a systematic review. Rev. Educ. Res. 90, 24–46. 10.3102/003465431987715338293548

[B27] Polanin J. R. Hennessy E. A. Tsuji S. (2020). Transparency and reproducibility of meta-analyses in psychology: a meta-review. Perspect. Psychol. Sci. 15, 1026–1041. 10.1177/1745691620906416 32516081

[B28] Polanin J. R. Pigott T. D. Espelage D. L. Grotpeter J. K. (2019). Best practice guidelines for abstract screening large-evidence systematic reviews and meta-analyses. Res. Synth. Methods 10, 330–342. 10.1002/jrsm.135426710217

[B29] Prensky M. (2001). Digital natives, digital immigrants part 2: do they really think differently? On the Horizon 9, 1–6. 10.1108/10748120110424843

[B30] Review Manager (2014). RevMan [Computer Program] (5.3) [Computer Software]. London: The Cochrane Collaboration.

[B31] Rogers E. M. (2003). Diffusion of Innovations, 5th Edn. New York, NY: Simon and Schuster.

[B32] Slavin R. E. (2008). Perspectives on evidence-based research in education-what works? issues in synthesizing educational program evaluations. Educ. Research. 37, 5–14. 10.3102/0013189X0831411738293548

[B33] Thomas J. Graziosi S. Brunton J. Ghouze Z. O'Driscoll P. Bond M. (2020). EPPI-Reviewer: Advanced Software for Systematic Reviews, Maps and Evidence Synthesis. EPPI-Centre Software. Available at: https://eppi.ioe.ac.uk/cms/Default.aspx?tabid=2967 (accessed October 18, 2023).

[B34] van de Schoot R. de Bruin J. Schram R. Zahedi P. de Boer J. Weijdema F.. (2021). An open-source machine learning framework for efficient and transparent systematic reviews. Nat. Mach. Intell. 3, 125–133. 10.1038/s42256-020-00287-7

[B35] Van der Mierden S. Tsaioun K. Bleich A. Leenaars C. H. C. (2019). Software tools for literature screening in systematic reviews in biomedical research. Altex 36, 508–517. 10.14573/altex.1902131 31113000

[B36] Wallace B. C. Small K. Brodley C. E. Lau J. Trikalinos T. A. (2012). “Deploying an interactive machine learning system in an evidence-based practice center: Abstrackr,” in Proceedings of the 2nd ACM SIGHIT International Health Informatics Symposium, 819–824.

[B37] Wang T. Cheng E. C. K. (2022). “Towards a tripartite research agenda: a scoping review of artificial intelligence in education research,” in Artificial Intelligence in Education: Emerging Technologies, Models and Applications. AIET 2021. Lecture Notes on Data Engineering and Communications Technologies, Vol 104, eds. E. C. K. Cheng, R. B. Koul, T. Wang, and X. Yu (Singapore: Springer), 16.

[B38] Wang Z. Nayfeh T. Tetzlaff J. O'Blenis P. Murad M. H. (2020). Error rates of human reviewers during abstract screening in systematic reviews. PLoS ONE 15:e0227742. 10.1371/journal.pone.0227742 31935267 PMC6959565

[B39] Zhang Q. Neitzel A. (2024). Choosing the right tool for the job: screening tools for systematic reviews in education. J. Res. Educ. Effect. 17, 513–539. 10.1080/19345747.2023.2209079

