# Supplementary material for: Evaluating the adoption of handsearching, citation chasing, and screening tools in education research: a survey study
Source: Front Res Metr Anal. 2024 Nov 27;9:1430355. doi: 10.3389/frma.2024.1430355 (PMC11632621; doi:10.3389/frma.2024.1430355)
Supplement: Supplementary file 1 [file Data_Sheet_1.docx]

**Supplemental Materials**

**Description of the tools included in the survey**

***Handsearching and Citation chasing tools***

Citationchaser

Citationchaser is an open R package created in 2022 to support researchers in backward and forward citation chasing. It is also available through a web-based Shiny app with a user-friendly interface. In the “article input” users can search one or more articles and ask for the reference list and/or the citations. The tool supports several article identifiers, such as DOIs, PMCIDs. The tool provides the number of records identified and the set of records to be downloaded as a RIS file. Citationchaser has the advantage to directly deduplicate the records, providing a unique set of records.

CoCite

CoCites developed by Janssens and Gwinn (2015) is a citation-based method for searching relevant scientific literature for a research synthesis. Based on one or more articles the tool searches and locates all the records in the reference list of the listed articles and the ones that cited those articles.

Paperfetcher

Paperfetcher a free and open-source Python package and an accompanying web-app. Paperfetcher automates the retrieval of article metadata for handsearching. With Paperfetcher’s assistance, researchers can retrieve article metadata from designated journals within a specified time frame in just a few clicks. In addition to handsearching, it also incorporates a beta version of citation searching in both forward and backward directions. Paperfetcher has an easy-to-use interface, which allows researchers to download the metadata of retrieved studies as a list of DOIs or as an RIS file to facilitate seamless import into systematic review screening software.

SpiderCite

SpiderCite is one of the tools included in the Systematic Review Accelerator (SRA) developed by the Institute for Evidence-based Healthcare. The suite of automation tools is designed to support various stages involved in the process of conducting a systematic review. The SRA tools aid in tasks such as citation search, screening, and compiling review results. SpiderCite is designed to perform citation chasing by automatically identifying and retrieving all citing and cited articles from a given set of studies that are uploaded.

Reference management tools

Reference managers are tools to manage bibliographic references and related materials (e.g., full texts in PDF). They help deduplication and the organization of the references after the comprehensive search in databases or other search strategies. Usually these tools support several formats, such as RIS, XLM, bibtex. Among them, we included in our survey Zotero, EndNote, Mendeley.

***Screening tools***

Abstrackr

Abstrackr is a free tool developed in early 2010 by Tufts Evidence-based Practice Center (Wallace et al., 2012) that semi-automates the screening process and enables teamwork. It includes private tags for blind review and supports the abstracts sorting in order of their probability of inclusion. The tool has several weaknesses, including lack of the possibility to detect and remove duplicate records. It employs support vector machine and shallow neural networks models that have limited modeling capacity.

ASReview

ASReview is a software developed in 2019 by researchers at Utrecht University (Van der Schoot et al., 2020). It is based on active machine learning models that predicts the relevance of studies starting from a limited number of instructions. The machine begins to gradually submit to reviewers only the studies that meet those criteria that meet their choices. The advantages of this tool concern the potential to identify up to 95% of the relevant studies by screening no more than 40% of the entire dataset, thus saving at least 60% of the time of a traditional screening (Ferdinands , 2020). The tool also has some limitations. It only allows the title and abstract screening phase and does not support simultaneous collaboration in the software between two or more reviewers. By the ASRreview relevancy ranking of records, reviewers have to arbitrarily decide when to stop reviewing and how many relevant studies to extract from the relevancy ranking.

Covidence

Covidence is a software created by an Australian non-profit organization in 2013 that supports title and abstract screening, full-text review, and data extraction phases. The online interface is simple and uses PRISMA review steps (Kohl et al., 2018). The tool has recently been equipped with a machine learning system that shows the records in order of relevance, based on ongoing screening. The main disadvantage of this software is that it is not for free, and the review is not completely blinded because the tool allows notes and tags.

DistillerSR

DistillerSR was developed by Evidence Partners Inc. from Canada. It is a well-known web-based tool that includes PubMed integration and an artificial intelligence component named DistillerAI. It is primarily suited for randomized clinical trials and includes a quality assessment feature to rate study quality. Additionally, it offers a "reviewer compatibility" feature that allows a junior and a senior reviewer to collaborate on reviewing studies and avoid having two junior reviewers evaluate the same study. However, the tool's limit is its cost, with subscriptions ranging from $15 to $75 per month for students, depending on the number of collaborators and projects.

EPPI-Reviewer

EPPI-Reviewer, which was launched in 2010 by the Social Science Research Unit at the Institute of Education, University College London and University of London, is designed for social science researchers. It employs machine learning to support the title and abstract screening and full-text review. This system has the potential to reduce the number of records to be screened in the range of 9% to 60% (Tso et al., 2020). However, the settings for screening (e.g. importing coding tools) require numerous steps and too many links between various sections of the software.

Rayyan

Rayyan is a web-based tool developed by the Qatar Computing Research Institute in 2014. Rayyan has a free version with basic functions as well as a paid version. It offers the possibility of shortening screening times through an active learning model based on the reviewers’ inputs. After 50 inputs, the system, through the support vector machine classifier model, evaluates the relevance of the studies that remain to be reviewed on the basis of the characteristics of the studies already reviewed (Ouzzani et al., 2016). The result is a ranking list of studies classified on a scale of 0 to 5 stars. This may save about 50% of the time usually spent in screening (Ouzzani et al., 2016). The tool has several limitations: the exclusive support of title and abstract screening; the review is not completely blinded because notes and tags are allowed; disagreements between users to include or exclude a record must be manually resolved through unblinded consensus.

RevMan

RevMan is one of the earliest tools available to researchers who conduct systematic reviews for screening and full-text review. It was developed in 2008 by the UK-based Cochrane Collaboration. While it is free for academic researchers, licensing is required for commercial use. However, according to research by Wang and Leeflang (2019), RevMan is not particularly user-friendly. Moreover, more complex models (e.g., Bivariate model) cannot be fitted in the tool.

Pallath, A., & Zhang, Q. (2022). Paperfetcher: A tool to automate handsearching and citation searching for systematic reviews. *Research Synthesis Methods*.

Haddaway, N. R., Grainger, M. J., & Gray, C. T. (2022). Citationchaser: A tool for transparent and efficient forward and backward citation chasing in systematic searching. *Research Synthesis Methods*, *13*(4), 533-545.

Cooper C, Booth A, Varley-Campbell J, Britten N, Garside R. Defining the process to literature searching in systematic reviews: a literature review of guidance and supporting studies. BMC Med Res Methodol. 2018;18(1):85. doi:10.1186/s12874-018-0545-3

Institute for Evidence-based Healthcare. SR-accelerator system- atic review accelerator published 2021. Accessed January 1, 2022. https://sr-accelerator.com/#/help/spidercite

**IRB**

Johns Hopkins University: IRB #HIRB00013268

University of Cagliari: IRB n. 0113396

University of Perugia: IRB n. 222024

**Recruitment**

Email invitation template

Dear [Insert Author's Name and Title Here],

We are a research team working at Johns Hopkins University (USA) and University of Cagliari and Perugia (Italy). Our team is in the process of collecting information on the screening tools currently used by systematic reviewers in the field of educational research internationally. We are offering educational researchers who conduct systematic reviews the opportunity to contribute their expertise and experience to our project through participating in this survey. By taking part, you will have the chance to inform the development of our work in a valuable way, and to help improve the use of software for title and abstract screening in educational reviews.

The survey consists of two sessions, the first one of 14 multiple choice questions and should take about ten minutes of your time, and the second one that consists of running a trial project with one of the software tools under evaluation and answering 19 close-ended questions and 11 brief open-ended questions. Your participation in this research study is voluntary.

We would appreciate if you could share that survey with your colleagues who are interested in educational systematic reviews and meta-analyses. Please reach out if you have any questions and we appreciate your time on this request.

https://unififorlilpsi.qualtrics.com/jfe/form/SV_2awjX268dIx7aya

Best Regards,

Amanda Neitzel (Inns)

Marta Pellegrini

Qiyang Zhang

Francesco Marsili

**Codebook**

Tools for Screening in Systematic Reviews

Start of Block: This survey aims to collect information on the screening tools currently used by

Q1.1 Hi there!

Out of all the possible clicks, we're happy you clicked here.

This survey was created by a team of passionate researchers from Johns Hopkins University (USA) and the Universities of Cagliari and Perugia (Italy). Your responses will help us understand the searching and screening tools currently used by systematic reviewers in education. The survey will take around 15 minutes to complete. We really appreciate you taking the time to complete it!

Your participation in this research study is voluntary. If you decide to participate in this research survey, you may withdraw at any time with no penalty. Your responses will be fully anonymized and confidential, so we hope you feel comfortable answering honestly. Data, without identifying information, will be made publicly available in an online database for re-analysis by other researchers. Results of this study may be presented at conferences or published in journals, books, and the popular media. Your answers will help future researchers to design more user-friendly tools!

If you have any general questions or concerns, please contact Amanda Neitzel, Deputy Director of Evidence Research and Research Scientist at the Center for Research and Reform in Education at Johns Hopkins University, at 410-616-2347 or aneitzel@jhu.edu. If you have questions or concerns specific to research in Asian regions, please contact Qiyang Zhang, Research Assistant at the Center for Research and Reform in Education, at qzhang74@jhu.edu. If you have questions or concerns specific to research in European regions, please contact Marta Pellegrini, Assistant Professor at the University of Cagliari, at marta.pellegrini@unica.it. This research has been reviewed according to the Homewood Institutional Review Board at Johns Hopkins University procedures for research involving human subjects.

End of Block: This survey aims to collect information on the screening tools currently used by

Start of Block: Searching and screening software

| Page Break |  |
| --- | --- |

hs First, we want to ask you some questions regarding handsearching tools.
Definition: Handsearching is a supplementary technique that reviewers often adopt to identify relevant studies, which involves systematically browsing through the tables of contents of a curated list of field-specific journals, abstracts, and conference proceedings.

hs1 How do you usually conduct handsearching?

o Manually browse through journals' or conferences' websites (1)

o Use certain tools (2)

o I don't conduct handsearching (3)

*Display This Question:*

*If How do you usually conduct handsearching? = Use certain tools*

hs2 What tool do you use?

________________________________________________________________

*Display This Question:*

*If How do you usually conduct handsearching? = Manually browse through journals' or conferences' websites*

hs3 How much time do you normally spend on manually conducting handsearching?

o Less than 10 hours (1)

o 10-20 hours (2)

o More than 20 hours (3)

cs Next, we want to ask you some questions regarding forward and backward citation searching tools.
Definition: Forward citation chasing searches for articles that cite a given article of interest while backward citation chasing searches for articles cited by the article of interest.

cs1 Which of the following citation searching tools have you heard of?

▢ Citationchaser (1)

▢ Paperfetcher (2)

▢ Sci-Finder (3)

▢ Web of Science (4)

▢ Scopus (5)

▢ Google Scholar (6)

▢ SpiderCite (7)

▢ Other: (11) __________________________________________________

cs2 Which one have you used the most?

o Citationchaser (1)

o Paperfetcher (2)

o Sci-Finder (3)

o Web of Science (4)

o Scopus (5)

o Google Scholar (6)

o SpiderCite (7)

o Other: (11) __________________________________________________

| Page Break |  |
| --- | --- |

cs3 Why is ${cs2/ChoiceGroup/SelectedChoicesTextEntry} the one you used the most?

________________________________________________________________

________________________________________________________________

________________________________________________________________

________________________________________________________________

________________________________________________________________

cs4 To what extent do you require technical support to be able to use ${cs2/ChoiceGroup/SelectedChoicesTextEntry}?

o Not at all (1)

o Slightly (2)

o Somewhat (3)

o Quite a bit (4)

o A tremendous amount (5)

cs5 How complex do you find ${cs2/ChoiceGroup/SelectedChoicesTextEntry}?

o Not complex at all (1)

o Slightly complex (2)

o Somewhat complex (3)

o Quite complex (4)

o Very complex (5)

cs6 How quickly do most people learn ${cs2/ChoiceGroup/SelectedChoicesTextEntry}?

o Not quickly at all (1)

o Slightly quickly (2)

o Somewhat quickly (3)

o Quite quickly (4)

o Very quickly (5)

cs7 How satisfied are you with ${cs2/ChoiceGroup/SelectedChoicesTextEntry}?

o Not satisfied at all (1)

o Slightly satisfied (2)

o Somewhat satisfied (3)

o Quite satisfied (4)

o Very satisfied (5)

cs8 What are some barriers stopping you from using the other tools? (e.g., ${cs2/ChoiceGroup/UnselectedChoices})

________________________________________________________________

________________________________________________________________

________________________________________________________________

________________________________________________________________

________________________________________________________________

| Page Break |  |
| --- | --- |

cs9 Rank the following searching strategies from most valuable to least valuable

______ Database search (1)

______ Handsearching (2)

______ Forward citation searching (3)

______ Backward citation searching (4)

cs10 Rank the following searching strategies from most time-consuming to least time-consuming

______ Database search (1)

______ Handsearching (2)

______ Forward citation searching (3)

______ Backward citation searching (4)

| Page Break |  |
| --- | --- |

st Next, we have some questions regarding screening tools.
Definition: Screening tools assist researchers to screen title and abstract as well as full text to determine eligibility.

st1 Which of the following have you heard of used for screening?

▢ Abstrackr (1)

▢ Covidence (2)

▢ ASReview (3)

▢ RevMan (4)

▢ Rayyan (5)

▢ EPPI-Reviewer (6)

▢ DistillerSR (7)

▢ Spreadsheet (i.e., Excel/Google Sheets) (8)

▢ Relational Database (i.e. Access/Filemaker) (9)

▢ Citation Management Program (i.e. Zotero/EndNote) (10)

▢ Other: (11) __________________________________________________

st2 Which one have you used the most for screening?

o Abstrackr (1)

o Covidence (2)

o ASReview (3)

o RevMan (4)

o Rayyan (5)

o EPPI-Reviewer (6)

o DistillerSR (7)

o Spreadsheet (i.e., Excel/Google Sheets) (8)

o Relational Database (i.e. Access/Filemaker) (9)

o Citation Management Program (i.e. Zotero/EndNote) (10)

o Other: (11) __________________________________________________

| Page Break |  |
| --- | --- |

st3 Why did you use ${st2/ChoiceGroup/SelectedChoicesTextEntry} the most?

________________________________________________________________

________________________________________________________________

________________________________________________________________

________________________________________________________________

________________________________________________________________

st4 To what extent do you require technical support to be able to use ${st2/ChoiceGroup/SelectedChoicesTextEntry}?

o Not at all (1)

o Slightly (2)

o Somewhat (3)

o Quite a bit (4)

o A tremendous amount (5)

st5 How complex do you find ${st2/ChoiceGroup/SelectedChoicesTextEntry}?

o Not complex at all (1)

o Slightly complex (2)

o Somewhat complex (3)

o Quite complex (4)

o Very complex (5)

st6 How quickly do most people learn ${st2/ChoiceGroup/SelectedChoicesTextEntry}?

o Not quickly at all (1)

o Slightly quickly (2)

o Somewhat quickly (3)

o Quite quickly (4)

o Very quickly (5)

st7 How satisfied are you with ${st2/ChoiceGroup/SelectedChoicesTextEntry}?

o Not satisfied at all (1)

o Slightly satisfied (2)

o Somewhat satisfied (3)

o Quite satisfied (4)

o Very satisfied (5)

st8 For what main functions did you use ${st2/ChoiceGroup/SelectedChoicesTextEntry} for?

▢ Duplication removal (1)

▢ Title & Abstract Screening (2)

▢ Full-text review (3)

▢ Data extraction (4)

▢ Other (5) __________________________________________________

st9 What are some barriers stopping you from using the other tools? (e.g., ${st2/ChoiceGroup/UnselectedChoices})

________________________________________________________________

________________________________________________________________

________________________________________________________________

________________________________________________________________

________________________________________________________________

| Page Break |  |
| --- | --- |

st10 Rank the following features from the most important to the least important

______ Bulk application (being able to download or upload in bulk) (1)

______ Machine learning (AI-automation) (2)

______ Collaboration (3)

______ Blind review (4)

______ Deduplication (5)

______ Inter-rater reliability report (6)

______ Research update (being able to come back and update prior reviews) (7)

______ Accessibility (cost) (8)

______ Process documentation (e.g., PRISMA diagram) (9)

st11 Is your review team currently using AI-automated functions at any stage of the systematic review?

o Yes (1)

o No, but plan to use in the future (2)

o No plan to use at all (3)

o Not sure (4)

*Display This Question:*

*If Is your review team currently using AI-automated functions at any stage of the systematic review? = Yes*

st12 What kind of AI-automated software did you use?

o Abstrackr (1)

o ASReview (2)

o Rayyan (3)

o EPPI-Reviewer (4)

o DistillerSR (5)

o RobotAnalysis (6)

o FASTREAD (7)

o Sysrev (8)

o Other: (9) __________________________________________________

*Display This Question:*

*If Is your review team currently using AI-automated functions at any stage of the systematic review? = Yes*

st13 At what stages of the review process have you used AI-automated software?

▢ Duplication removal (1)

▢ Title & Abstract Screening (2)

▢ Full-text review (3)

▢ Data extraction (4)

▢ Other (5) __________________________________________________

*Display This Question:*

*If Is your review team currently using AI-automated functions at any stage of the systematic review? = No, but plan to use in the future*

*Or Is your review team currently using AI-automated functions at any stage of the systematic review? = No plan to use at all*

st14 What are the reasons that you haven’t started using an AI-supported systematic review process?

▢ Lack of information if they are available (1)

▢ Compatibility with current work flow (2)

▢ Access (3)

▢ Complexity of the process is intimidating (4)

▢ Lack of trust and confidence (5)

▢ Lack of time to figure out and understand the tools (6)

▢ Other: (7) __________________________________________________

End of Block: Searching and screening software

Start of Block: Demo

de Well done! We are almost there! In this last section, we want to get to know a bit about you.

de1 Have you ever conducted systematic reviews or meta-analyses in education?

o Yes (1)

o No (2)

de2 How many years have you conducted systematic reviews or meta-analysis in education?

o 0-2 (12)

o 2-4 (13)

o 4-6 (14)

o 6 or more (15)

de3 With approximately how many systematic reviews or meta-analysis projects have you been involved with?

o 0 (4)

o 1-2 (1)

o 3-4 (2)

o 5 or more (3)

de4 In which continent are you mostly conducting research in?

o North America (398)

o South America (399)

o Asia (400)

o Europe (594)

o Africa (595)

o Australia (596)

o Antarctica (597)

Q2.5 What is your current position?

o Undergraduate or Masters student (2)

o Doctoral student (1)

o Post-doc (3)

o Faculty (Assistant) (4)

o Faculty (Associate or Full) (5)

o Non-faculty research position (9)

o Non-university research position (10)

o Other: (7) __________________________________________________

gender What is your gender?

o Male (1)

o Female (2)

o Other (3) __________________________________________________

o Prefer not to answer (4)

age How old are you

o Under 18 (1)

o 18-24 years old (2)

o 25-34 years old (3)

o 35-44 years old (4)

o 45-64 years old (5)

o Above 64 (6)

End of Block: Demo

Start of Block: Block 3

end If you have any general questions or concerns, please contact Amanda Neitzel, Deputy Director of Evidence Research and Research Scientist at the Center for Research and Reform in Education at Johns Hopkins University, at 410-616-2347 or aneitzel@jhu.edu. If you have questions or concerns specific to research in Asian regions, please contact Qiyang Zhang, Research Assistant at the Center for Research and Reform in Education, at qzhang74@jhu.edu. If you have questions or concerns specific to research in European regions, please contact Marta Pellegrini, Assistant Professor at the University of Cagliari at marta.pellegrini@unica.it. This research has been reviewed according to the Homewood Institutional Review Board at Johns Hopkins University procedures for research involving human subjects.

 If you are interested in any of the tools we mentioned in this survey, here are the links for searching tools:
 [Paperfetcher](https://share.streamlit.io/paperfetcher/paperfetcher-web-app/main/paperfetcher_app.py): handsearching, citation searching
 [Citationchaser](https://estech.shinyapps.io/citationchaser/): citation searching
 [Google scholar, Scopus, Web of Science](https://lancaster.libguides.com/Litsearch/cited): citation searching
 [SpiderCite:](https://sr-accelerator.com/#/) citation searching

 Here are the links for screening tools:
 [Abstrackr](http://abstrackr.cebm.brown.edu/account/login) [Covidence](https://www.covidence.org/) [ASReview](https://asreview.nl/) [Rayyan](https://www.rayyan.ai/) [EPPI-Reviewer](https://eppi.ioe.ac.uk/CMS/Default.aspx?alias=eppi.ioe.ac.uk/cms/er4&amp;) [DistillerSR](https://www.evidencepartners.com/products/distillersr-systematic-review-software) [RevMan](https://revman.cochrane.org/#/myReviews)

End of Block: Block 3
